# Interspecific Hybridization Enhanced Tolerance to Salinity and Cadmium Stress Through Modifying Biochemical, Physiological, and Resistance Gene Levels, Especially in Polyploid Rice: A Sustainable Way for Stress-Resilient Rice

**DOI:** 10.1186/s12284-025-00776-6

**Published:** 2025-03-22

**Authors:** Lixia Sun, Fozia Ghouri, Jiacheng Jin, Minghui Zhong, Weicong Huang, Zijun Lu, Jinwen Wu, Xiangdong Liu, Muhammad Qasim Shahid

**Affiliations:** 1 State Key Laboratory for Conservation and Utilization of Subtropical Agro-Bioresources, Guangdong Laboratory for Lingnan Modern Agriculture, South China Agricultural University, Guangzhou, 510642 China; 2 Guangdong Provincial Key Laboratory of Plant Molecular Breeding, South China Agricultural University, Guangzhou, 510642 China; 3 Guangdong Base Bank for Lingnan Rice Germplasm Resources, College of Agriculture, South China Agricultural University, Guangzhou, 510642 China

**Keywords:** Transcription factors (TFs), Hybrid vigor, Polyploidy, Cadmium, Salt tolerance

## Abstract

Polyploid plants exhibit strong resistance to salt and cadmium (Cd) stress, which can adversely affect their growth, reducing crop quality and yield. Transcriptome analysis, antioxidant enzymatic activities, physiological measurements of reactive oxygen species, and heterosis analysis were performed on hybrids with neo-tetraploid rice and its progenitors. The results showed that diploid hybrids had fluctuating yields in early and late seasons, while tetraploid hybrids had consistent grain yield throughout. Transcriptome analysis revealed that gene expression related to sugar metabolism processes increased in tetraploid hybrids. Transcriptome analysis revealed several genes associated with heterosis and stress, including *OsEAF6*, which is associated with heterosis, and *OsCIPK14*, which is involved in defense signalling pathways. Furthermore, compared to the parents, hybrids have a much higher number of genes associated with abiotic stress. Consequently, diploid and tetraploid hybrids were treated with Cd (0 and 100 µM) and NaCl (200 mM) in the present study. Under Cd toxicity, the levels of carotenoids were reduced by 33.31% and 45.59%, while the levels of chlorophyll a declined by 16.00% and 27.81% in tetraploid and diploid hybrids, respectively, compared to the control. Tetraploid hybrids had the highest germination rate under salt stress and the lowest Cd uptake compared to diploid hybrids and their parents. In general, the activities of antioxidant enzymes exhibited a considerable drop, whereas the levels of H_2_O_2_ and MDA showed a remarkable increase in parents compared to hybrids. Under cadmium toxicity, the expression of *OsERF1* in tetraploid rice was increased, and *OsABCC1* and *OsHMA3* were highly expressed in neo-tetraploid rice. Interspecific hybrid (*indica* and *japonica*) displayed enhanced tolerance to cadmium and salinity stress, potentially serving as a natural resource to improve rice resilience. These findings provide a basis for understanding polyploid rice’s gene expression pattern, environmental tolerance, and heterosis.

**Supplementary Information:**

The online version contains supplementary material available at 10.1186/s12284-025-00776-6.

## Introduction

The climate is changing dramatically, and the trend of global warming is accelerating. Therefore, crop breeding is very important to cope with climate change. Conversely, polyploid is considered more environmentally stable than diploid (Ramsey 2011). The advantages of polyploid rice are mostly characterized by robust growth, strong stress resistance, large grains, large biomass, and high nutrient content (Chen et al. 2022; Guo et al. 2017; Liu and Sun 2017; Yu et al. 2020, 2021). At present, research on rice polyploidy mainly focuses on tetraploid rice. From the perspective of chromosomes, tetraploid rice can be divided into autotetraploid rice and allotetraploid rice. Tetraploid rice has greater vegetative organs and biomass, and there are differences in the utilization of heterosis from diploid traits (Shahid et al. 2011). Allotetraploid rice mainly has non-domesticated characteristics, which are challenging to produce and apply. Previous studies have shown that allotetraploid rice can be rapidly domesticated by using gene editing technology (Yu et al. 2021). Concerning autotetraploid rice, the low seed setting rate was the main problem, and the embryo fertility was significantly lower than that of diploid rice, and the tetraploid hybrids enhanced the accumulation of pollen sterility loci (Shahid et al. 2010; Wu et al. 2015a). However, the presence of some neutral gene loci (*Sa-n*) plays an important role in positive heterosis (Chen et al. 2019). The emergence of meiotic stable polyploid rice (PMeS rice), tetraploid rice of the Haitian series (HT4), and neo-tetraploid rice has overcome autotetraploid rice’s low seed setting rate (Ghaleb et al. 2020; He et al. 2011; Zhang et al. 2024). Neo-tetraploid and PMeS rice lines have the potential to exhibit fertility rates surpassing 70%, while the seed setting rate of their F_1_ hybrids can also reach 70% (Ghaleb et al. 2020).

Heterosis, also known as hybrid vigor, can be observed not only in diploid species but also in the 1 N (haploid) stage of polyploid plants, including gametes and gametophytes composed of pollen and oocysts (Butruille and Boiteux 2000; Groose and Bingham 1991). Polyploid heterosis frequently exhibits greater vigor than its diploid progenitors, and multiple chromosomal groups in polyploids increase the likelihood of gene redundancy (Comai 2005). The dosage effect of heterosis is well recognized, and an important factor in heterosis is the accumulation of a high number of rare superior alleles with positive heterosis (Huang et al. 2015). Altering the amplitude frequency of the biological clock while maintaining the rhythm is a potential strategy to enhance the heterosis of hybrids and allotetraploids (Chen 2010). The F_1_ hybrids exhibited significant heterosis, with distinct expression patterns observed in genes associated with meiosis, glucose metabolism, and starch synthase during various developmental stages of polyploid rice (Chen et al. 2019). Hybridization yielded high fertility hybrid, and the advantages could be preserved for several generations (Chen et al. 2022). So, it is crucial to study the heterosis of tetraploid rice (Liu et al. 2023a, b). Moreover, previous studies have shown that autotetraploid rice has a greater tolerance against heavy metals than diploid rice (Ghouri et al. 2024). Therefore, it will be interesting to investigate the performance of diploid and tetraploid rice hybrids against abiotic stress. *Indica*-*japonica* hybridization has a more substantial advantage over diploid *indica*-*japonica* hybridization (Hu et al. 2010).

Reducing plant abiotic stresses can increase plant productivity (Mahmud et al. 2023). Under salt stress, the induction and expression levels of stress response genes, including the JA pathway, were higher in tetraploid rice than in diploid rice (Wang et al. 2021). Heterohexaploid wheat microRNAs provide better salt tolerance in polyploidy than diploid ancestors (Liu and Sun 2017). The resistance to salt and the harm caused by heavy metal stress vary significantly among various rice varieties. Under Cd stress, plants’ root and shoot growth and development were affected (Liu et al. 2023a, b). Varieties with high hemicellulose content in the rice roots’ cell wall limit Cd transport from buds to grains (Liu et al. 2016). Studies have demonstrated that tetraploid plants possess a notable edge when withstanding the effects of heavy metal stress (Ghouri et al. 2023a, b). Changes in organic acids and amino acids in root exudates affect cadmium uptake by rice roots (Fu et al. 2018). Cadmium-tolerant varieties significantly changed proteins related to carbon metabolism, proteolytic enzymes, some transcription factors, and RNA helicases (Fang et al. 2019). Our previous studies on autotetraploid rice found that *Sa*, *Sb*, and *Sc* pollen sterility loci can cause autotetraploid *indica/japonica* hybrid sterility (Wu et al. 2015b, 2019), which could be overcome by double neutral genes, *Sa*^*n*^ and *Sb*^*n*^ (Wu et al. 2017). Multiple genes regarding salt tolerance have been identified, such as *miR396b*/*GRF6*, which regulates salt tolerance in rice by regulating changes in antioxidant enzyme activity (Yuan et al. 2024). *OsLHY* (LATE ELONGATED HYPOCOTYL) can enhance the salt tolerance of rice by modulating Na^+^/K^+^ homeostasis and ABA signalling (Li et al. 2024).

Several strategies have been the focus of research efforts to reduce Cd accumulation in rice grains, including breeding Cd-tolerant rice varieties, soil amendments to lower the availability of Cd, and genetic engineering to modify the function or expression of metal transporters like *OsABCC1*,* OsHMA3*, and *OsNRAMP1*. This study aimed to (1) compare the effects of hybridization on diploid and tetraploid rice at morphological, cytological, and molecular levels; (2) assess heterosis between intraspecific and interspecific diploid and tetraploid rice hybrids; and (3) evaluate the impacts of cadmium and salinity on diploid and tetraploid rice hybrids and their parental lines. Here, we generated hundreds of hybrid combinations and selected one pair of *indica* and *indica* (H3 × P1) hybrid and one pair of *indica* and *japonica* hybrid (H3 × P2) through comprehensive phenotypic analysis. We examined the influence of polyploidy on adaptation to environmental stress through experiments incorporating salinity and Cd stress, yielding significant genetic resources for rice breeding. This comprehensive study evaluated how well rice performed in terms of hybridization and polyploidy from various morphological parameters to RNA-seq and several physiological metrics under salt and cadmium stress.

## Results

### Heterosis Evaluation of P1-2x × H3-2x and P1 × H3 Hybrids

In the F_1_ generation, the total number of grains in diploid hybrid D1 (P1-2x × H3-2x) was approximately 1900, 1.6 times higher than the commercial variety P1-2x, which had 1478 grains. The seed setting rate was 76.07%, and grain length, grain width, main panicle length, number of effective tillers per plant, 1000-grain weight, yield per plant, and biomass per plant were 8.12 mm, 2.91 mm, 24.69 cm, 8, 20.82 g, 32.84 g, and 24.02 g, respectively (Table S1a).

Compared to the commercially available P1-2x, hybrid D1 exhibited advantages in total grain number, filled grain number, grain width, yield per plant, flag leaf width, plant height, main panicle length, effective tillers, biomass per plant, and 1000-grain weight. The strongest advantages were observed in total grain number and yield per plant, which were 60.12% and 62.41% higher than commercial cultivar. The mid-parent heterosis analysis revealed positive heterosis for total grain number, filled grain number, seed setting rate, grain width, yield per plant, flag leaf width, plant height, main panicle length, effective tiller, biomass per plant, and 1000-grain weight. Specifically, the total and filled grain numbers had 90.30% and 88.31% advantages (MPH), respectively, while the advantage in yield per plant was over 100%. Super-parent and average-parent heterosis analysis showed superior total grain number, filled grain number, and yield per plant. The D1 hybrid had non-significant advantages over its parents in grain length and seed setting rate, but it had greater yield per plant due to improvements in total grain number and filled grain number. Overall, diploid rice exhibited stronger heterosis than the control cultivar (Table S1b).

Tetraploid rice hybrid T1 (P1 × H3) was created through crossing of P1 (*indica*) with H3, also known as neo-tetraploid rice, which our research group generated through the directional selection of autotetraploid rice. H3 was then hybridized with the autotetraploid rice line, P1, to produce a T1 hybrid. T1 has a total grain number of about 861, 678 filled grains, a seed setting rate of 78.71%, a grain length of 10.36 mm, a grain width of 3.13 mm, a main panicle length of 28.44 cm, six effective tillers, the 1000-grain weight of 33.12 g, yield per plant of 22.85 g, and biomass of 25.95 g (Table S1c). Among the key yield indicators, the advantage (SPH) of T1’s 1000-grain weight is 60.26%, and all other characters except for the total grain number and filled grain number exhibit obvious advantages. The mid-parent heterosis for T1 showed that the total grain number, filled grain number, and yield per plant increased to 98.74%, 126.28%, and 206.25%, respectively. Over-parent and average heterosis indicated that the yield per plant was more than 100% (Table S1d). However, compared to the commercial variety P1-2x, D1 has advantages over T1 regarding total grain number, filled grain number, seed setting rate, and effective panicle number. T1 has stronger grain length, width, and biomass advantages than D1, but increasing the number of filled grains is crucial for further yield improvement. Overall, both hybrids exhibit high heterosis, and the diploid performs better than the tetraploid.

### Heterosis Evaluation of P2-2x × H3-2x and P2 × H3 Hybrids

**Fig. 1:**
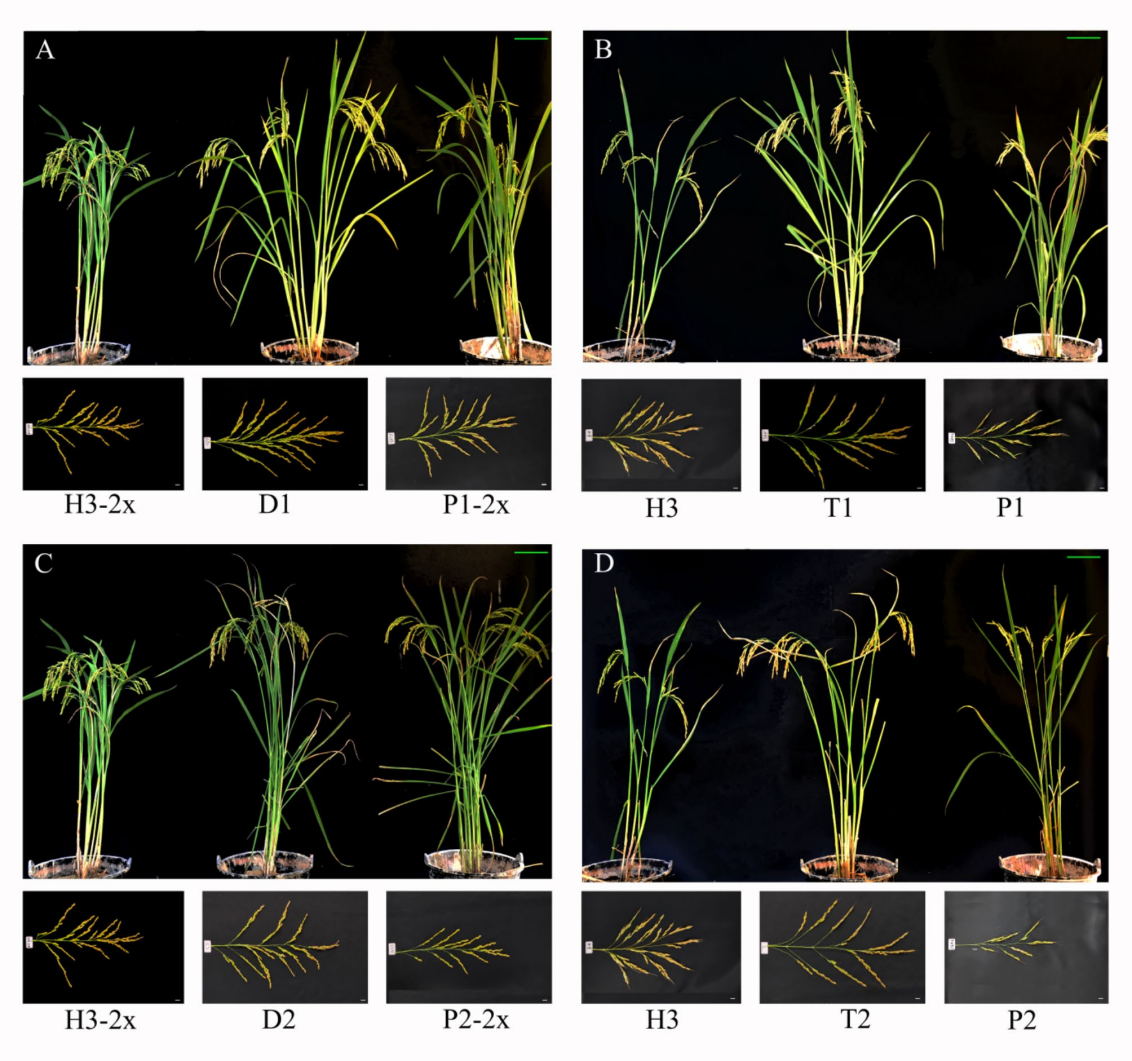
F_1_ hybrids generated by crossing H3 (neo-tetraploid) with typical *indica* and *japonica* rice cultivars. H3-2x is a reverted diploid rice of H3; P1 is a tetraploid *indica* rice variety; P1-2x is a diploid *indica* rice variety; D1 (P1-2x × H3-2x); and T1 (P1 × H3); P2 is a tetraploid *japonica* rice variety; P2-2x is a diploid *japonica* rice variety; D2 (P2-2x × H3-2x); and T2 (P2 × H3). Green Scale bar: 20 cm, White Scale bar: 5 mm

It can also be seen from the plant morphology that hybrids have obvious heterosis (Fig. 1A, B, C and D). The D2 diploid hybrid (P2-2x × H3-2x) has a total grain number of approximately 1459, with around 587 filled grains higher than one of the parents. However, the seed setting rate was 31.1-44.42% lower than the parent, while the yield per plant was 11 g, around 3.3 g higher than the parent (Table S2a). The total number of grains and filled grain numbers showed competitive, mid-parent, and super-parent advantages (Table S2b).

Tetraploid T2 (P2 × H3) hybrid was generated by crossing P2 (autotetraploid), which exhibits a poor seed setting rate with high fertility H3 (neo-tetraploid rice). The T2 hybrid exhibited the following characteristics: an approximate total of 722 grains, of which 540 were filled, a seed setting rate of 74.48%, and a yield per plant of 19.97 g (Table S2c). Compared to the commercial variety P2-2x, T2 has over-standard parent advantage (SPH) in grain width and 1000-grain weight, which were 55.22% and 67.34%, respectively. The mid-parent heterosis, over-parent heterosis, and average heterosis suggest that the yield per plant can reach 100% or more heterosis (Table S2d). The yield per plant of the tetraploid hybrid was higher than that of the diploid hybrid, mainly due to the lower grain number of D2, resulting in a lower yield. The low grain number may be due to the *indica*-*japonica* hybrid sterility, but tetraploids can relatively overcome this sterility, providing ideas for studying *indica* and *japonica* hybrid sterility.

The mean results during the early and late seasons display differences in agronomic traits due to the varying temperatures. Notably, the early season of hybrids outperforms the late season in crucial yield traits like total grain number, filled grain number, seed setting rate, and yield per plant. The late season of parents, in contrast, exceeds the early season. However, the fluctuation in tetraploid hybrids during both seasons is lower than that of diploid hybrids. Regarding hybrids, namely D2 and T2, tetraploid hybrids exhibited significantly higher grain length, grain width, flag leaf width, and 1000-grain weight than diploid hybrids. Observations of pollen and embryo sac fertility were made to explore the low seed setting rate of P1 and P2 preliminarily, which showed that pollens of both lines developed normally (Figure S1). The embryo sac fertility of P1 and P2 was 47.26% and 44.13%, respectively (Figure S2). During P1 and P2’s embryo sac development, the mature embryo sacs failed to fertilize and develop into pre-embryo normally, with an enlarged embryo sac cavity and polar nuclei.

### Dominance and Epistatic Effects Contribute to Hybrid Vigor

We used two different hybrids (*indica* × *indica* and *indica* × *japonica*) to do the transcriptome analysis to analyze the expression levels of the genes. It also tries to identify the more critical stage by sampling at multiple stages. The spikelets were found to change the most at 5 days after fertilization. Transcriptome analysis of 114 samples in this group yielded 979.44 Gb of clean data. The clean data of all samples was 5.78 Gb, with a Q30 base percentage of 93.25% or above (Table S3). The efficiency of sequencing data was evaluated by comparing the proportion of mapped reads to clean reads, which directly reflects the utilization rate of transcriptome data. This comparison efficiency was used to determine whether the selected reference genome assembly was suitable for analysis. Valid data were evaluated using HISAT2 software and compared to the IRGSP reference genome. The comparison efficiency of reads and reference genome of each sample ranged from 92.76 to 98.63% (Table S4). Data with a correlation between duplicate samples of less than 0.8 were removed from the analysis (Figure S3).

**Fig. 2:**
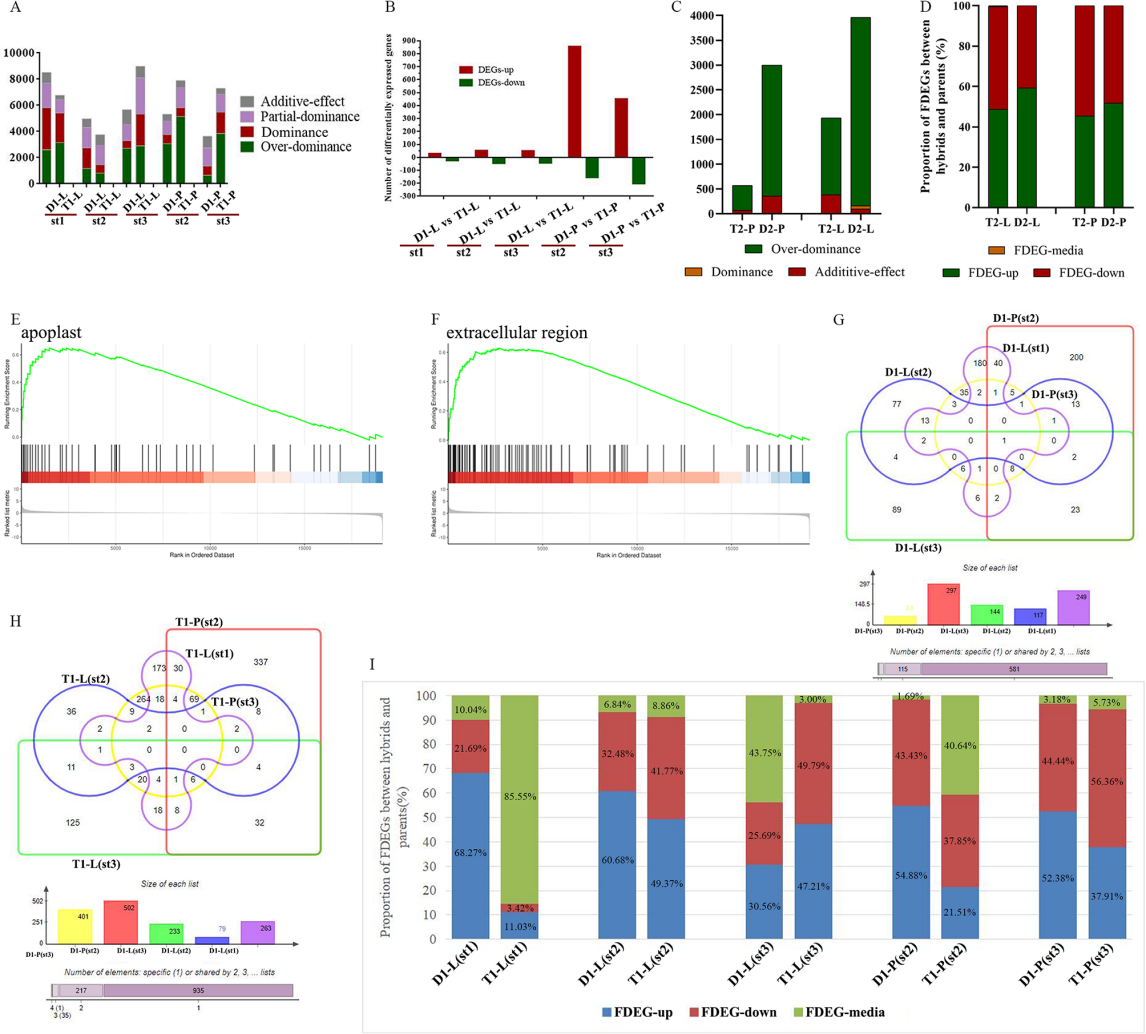
Comprehensive transcriptome analysis of D1, T1, D2, and T2 hybrids. (**A**) The effect analysis of D1 and T1 with their parents’ different genes, D1-L is the flag leaf of D1, D1-P is the panicle of D1, st1 means microspore stage, st2 means heading stage, and st3 means 5 days after fertilization; (**B**) The number of differentially expressed genes in the same tissues of D1 and T1 at the same time; (**C**): The effect analysis of D2 and T2 with their parents’ different genes; (**D**): Differentially expressed genes of FDEGs; (**E**-**F**) GSEA analysis of five different gene sets of D1 vs. T1; (**G**) Venn diagram of DEGFu-sp (D1), (**H**) Venn diagram of DEGFu-sp (T1); (**I**) Differentially expressed genes of FDEGs

The majority of gene sets that differ between hybrids D1 and T1 and their parents exhibited a dominant effect, while non-additive effects contributed to a lesser proportion. An analysis of the spikelet transcriptome at the heading stage and five days after fertilization revealed that the number of differential gene sets of D1 was lower than that of T1 (Fig. 2A), suggesting that the gene changes underlying heterosis are primarily due to the interaction of multiple genes and not simple additive values. Additionally, the number of gene changes in diploid hybrid D1 was relatively small and stable after the transition from vegetative growth to reproductive development, potentially explaining why D1 exhibits greater heterosis than T1. The number of differential genes in diploid hybrids vs. tetraploid hybrids (D1 vs. T1) during different flag leaf stages was less than 200 (Fig. 2B), indicating non-significant difference between the two regarding crop source.

After that, we focused on the samples 5 days after heading for D2 and T2. In the T2, half of the up-regulated and down-regulated genes were in FDEGs. In D2, 50-60% of the genes showed up-regulation. In the D2 and T2 hybrids, there were few genes between the two parental expressions (Fig. 2C). The analysis of the effects between D2 and T2 and the parents found that most were over dominant, a small part of the additive effect, and there was no partial-dominance and dominance (Fig. 2D). There were many up-regulated genes among the differential gene sets between D1 vs. T1 (spikelets at heading date and 5days after fertilization stage), most of these genes related to extracellular bodies and extracellular regions (Fig. 2E and F). Transcriptome analysis of T1 at different developmental stages showed that there were more common genes between different stages. However, D1 has fewer common genes at different stages than T1 (Fig. 2G and H). In the FDEG (differentially expressed functional genes between F_1_ and parents) gene set, the up-regulated genes of D1 hybrids were more than those of T1 hybrids except for the 5 days after fertilization (Fig. 2I). Some of these genes were selected for qPCR verification, which was consistent with the transcriptome results (Figure S4).

### Transcriptome Analysis Revealed High Abiotic Resistance in *Indica* × *Japonica* Rice Hybrid

**Fig. 3:**
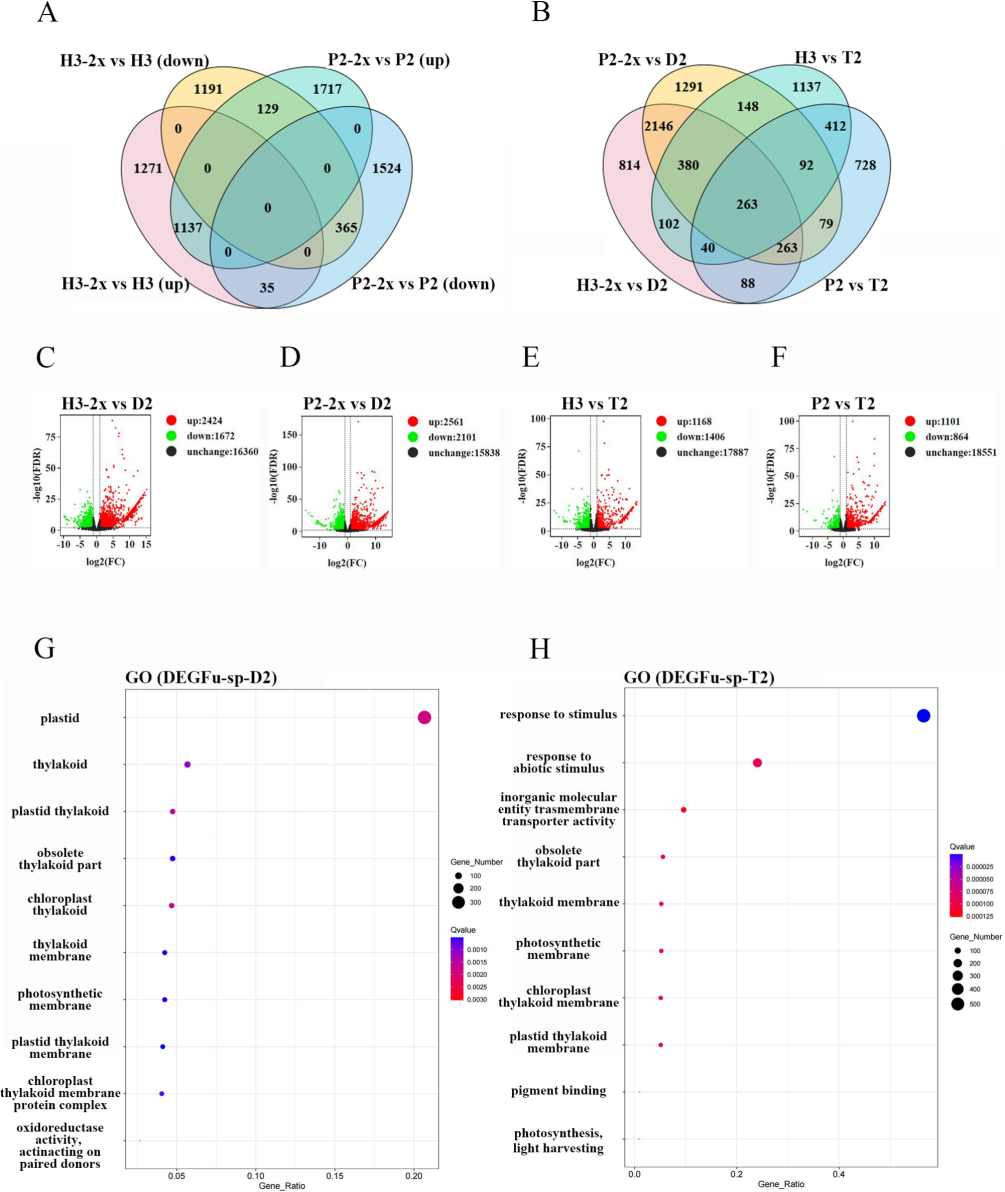
The number of differentially expressed genes and enrichment pathways between D2 and T2 (flag leaf at 5 days after fertilization) and their parents. The sampling period is the flag leaf 5 days after fertilization, and the Fold Change ≥ 2 and FDR < 0.01 are used as the screening criteria for differential genes. (**A**) Differentially expressed genes between tetraploid and its corresponding diploid; (**B**) Venn map of different genes between D2 and T2 and their parents; (**C**-**F**) The number of up-regulated genes between D2 and T2 and their parents; (**G**) GO enrichment map of the differential gene set between D2 and its parents; (**H**) GO enrichment map of the differential gene set between T2 and its parents

**Fig. 4:**
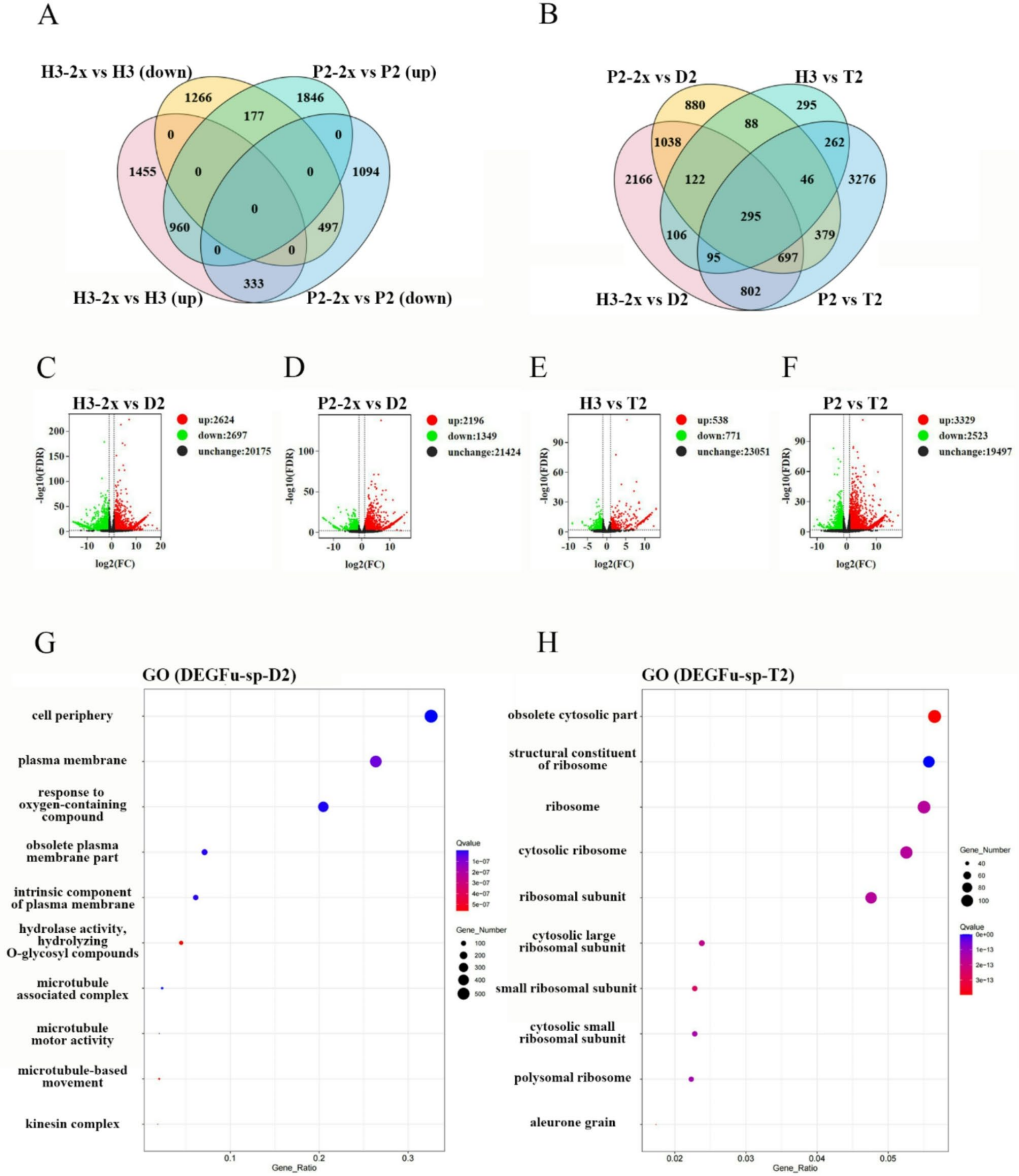
The number of differentially expressed genes and enrichment pathways between D2 and T2 (panicle at 5 days after fertilization) and their parents. The samples were collected from the spikelets 5 days after fertilization, and the Fold Change ≥ 2 and FDR < 0.01 are used as the screening criteria for detecting differential genes. (**A**) Differential genes between tetraploid and its corresponding diploid; (**B**) Venn map of different genes between D2 and T2 and their parents; (**C**-**F**) The number of up-regulated genes between D2 and T2 and their parents; (**G**) GO enrichment map of the differential gene set between D2 and its parents; (**H**) GO enrichment map of the differential gene set between T2 and its parents

The flag leaf and spikelet of the D2 (P2-2x × H3-2x) hybrid, 5 days after fertilization, were analyzed for their transcriptome sequencing. The analysis revealed that the hybrid was enriched in the environmental response pathway. D2 (P2-2x × H3-2x) is a hybrid of *indica* rice (H3-2x) and *japonica* rice (P2-2x), and T2 (P2 × H3) is a tetraploid hybrid after genome doubling. For source, there was a significant distinction between diploid and tetraploid rice hybrids, but a lesser difference between the tetraploid hybrids and their parental lines. Two distinct gene sets of hybrids D2 (P2-2x × H3-2x) and T2 (P2 × H3), along with their progenitors, exhibited an overlap of 263 genes, which may contribute greatly to the hybridization of *indica* and *japonica* rice (Fig. 3). The GO enrichment results of DEGs (Parents vs. D2 (P2-2x × H3-2x) about flag leaf at 5 days after fertilization) showed that the differential genes were active in plastids, chloroplast thylakoids, and other organelles, contributing to photosynthesis and reproductive development. In contrast, the GO enrichment results of DEGs (Parents vs. T2 (P2 × H3) about flag leaf at 5 days after fertilization) indicated that these differential genes were active in chloroplasts and responded to stress reactions, such as abiotic stress response. In addition, the results of spikelet transcriptome on the fifth day after fertilization showed that there were 3545 and 5321 different genes between hybrid D2 (P2-2x × H3-2x) and female and male parents, respectively (Fig. 4C and D). There are 5852 and 1309 differential genes between T2 (P2 × H3) and its maternal and paternal parents, respectively (Fig. 4E and F). Furthermore, 960 genes were up-regulated after the doubling of parents (Fig. 4A), and 295 genes were found to be common between different gene sets of hybrids D2 (P2-2x × H3-2x) and T2 (P2 × H3) and parents (Fig. 4B). The GO enrichment results of DEGs (Parents vs. D2 (P2-2x × H3-2x) panicle at 5 days after fertilization) showed that the differential genes were related to the cell periphery, plasma membrane, and microtubule association complex, responding to oxygenated compounds (Fig. 4G). On the other hand, the GO enrichment results of DEGs (Parents vs. T2 (P2 × H3) about panicle at 5 days after fertilization) suggested that obsolete cytosolic part, structural components of ribosomes, and aleurone particles were related to them (Fig. 4H). Some genes were selected for qPCR verification, and the results were authentic and reliable (Figure S5).

DEGFu-sp-D2 was expressed mainly at the transcriptional level as a receptor kinase in response to signal pathway and protein degradation during biological stress and was involved in abiotic and biological stress processes. Disease-related proteins mainly were increased, with positive responses to plant hormones, signal transduction, secondary metabolism, and abiotic stress. In flag leaf tissue, most of the genes related to secondary metabolism and cell wall were up-regulated, while in spikelets, most were down-regulated. Heat shock proteins were significantly up-regulated in both tissues, and transcription factors played a significant role in heterosis. The genes of ERF, bZIP, WRKY, MYB, and DOF families of transcription factors showed significant changes (Figure S6A, S6B, S6C, S6D). Unlike their parents, diploid hybrids were more resistant to disease and abiotic stress and sensitive to signal transduction.

The transcriptome analysis of the flag leaf and spikelet of hybrid T2 (P2 × H3), 5 days after fertilization, showed that the number of gene changes in cell response was less than that of diploid hybrids. The genes related to cell wall growth decreased in flag leaves but increased in spikelets, which was contrary to D2 (P2-2x × H3-2x) hybrids. D2 (P2-2x × H3-2x) hybrid compared to their parents, genes involved in plant hormones, cell wall growth, secondary metabolism, and abiotic stress were up-regulated (Figure S6E, S6F, S6G, S6H). In conclusion, compared to the parental diploid hybrids, tetraploid hybrids show significant advantages in environmental response and sensitivity to signal transmission. To explore the relationship between tetraploid hybrids and the environment/abiotic resistance, *indica*-*japonica* hybrids were further analyzed.

### Increased Abiotic Resistance of *Indica* × *Japonica* Hybrid Rice

**Fig. 5:**
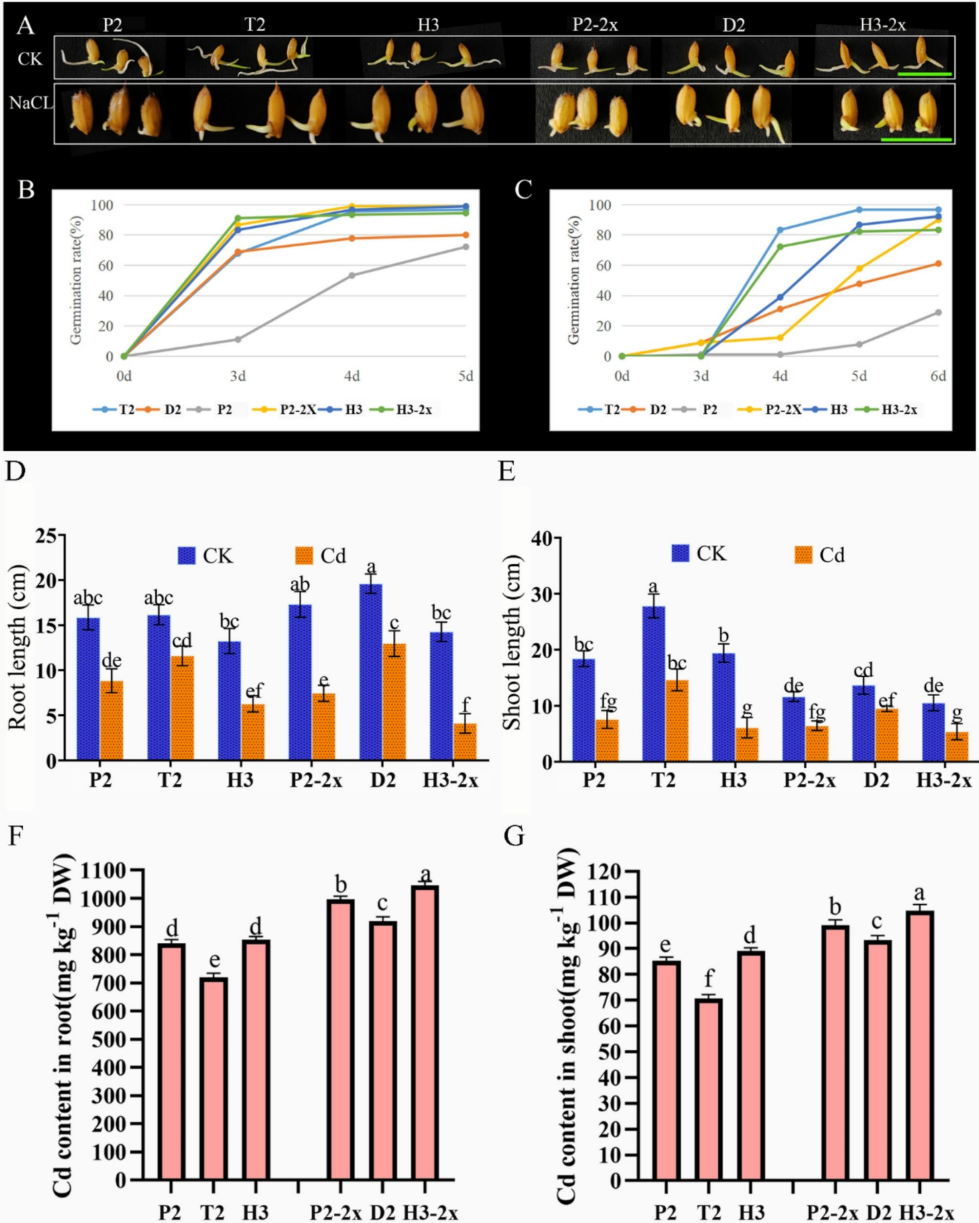
Germination rate of hybrids under salt stress and plant growth status of F_1_ hybrids and their parents under cadmium stress. (**A**) The figure shows the bud length of the hybrid and its parent; (**B**) The germination rate of hybrids and their parents under control treatment; (**C**) The germination rate of hybrids and their parents under NaCl treatment. The NaCl concentration was 200 mM, and the green scale is 1 cm; 0d: 0 days for treatment; 3d: processing for 3 days; 4d: processing for 4 days; 5d: 5 days for processing; 6d: 6 days for processing; (**D,E**) The root length and shoot length of plants under different treatments; (**F,G**) The Cd content in the roots and shoots of hybrids and their parents after Cd treatment. The concentration of CdCl_2_ was 100 µM. CK and Cd represent control and Cd treatments. H3 is neo-tetraploid rice; H3-2x is reverted diploid rice; P2 is a tetraploid *japonica* rice; P2-2x is a diploid *japonica* rice; D2 (P2-2x × H3-2x); and T2 (P2 × H3)

We selected two hybrids and their parents with high yield for further comparative studies. The seeds were subjected to salt stress treatment, and it was observed that the hybrids exhibited a germination rate of approximately 15% on the third day (Fig. 5A). By the fifth day, the germination rates of the hybrids ranged between 50% and 100%. On the other hand, the parents, with the exception of H3 and H3-2x, displayed germination rates ranging from 0 to 60% (Figs. 4C and 5B). By the sixth day, the hybrids were able to reach a germination rate of more than 80%, which revealed that hybrids were more salt-tolerant than their parents. Under Cd stress, a tetraploid hybrid was significantly more tolerant than the parent (Figure S7), and the maternal plants were more tolerant than the paternal plants. The hybrids had better growth than their parents under salt stress at the seedling stage (Figure S8). The shoot lengths of both hybrids exceeded those of the parents. The root of T2 is considerably longer than that of the parents, while the root of D2 exceeds the length of one parent.

Similarly, the diploid hybrid exhibited better growth than both parents under cadmium stress. Therefore, in order to further explore the reason for the increased tolerance, some phenotypic and physiological data were measured. The contribution of the data was estimated by effect size, PCA (principal component analysis), and correlation analysis. By partial Eta analysis, all indicators had high effects (Table S5). The correlation between the indexes was strong, and cadmium in roots and shoots was positively correlated with H_2_O_2_ (hydrogen peroxide) and MDA (malondialdehyde), and negatively correlated with chlorophyll, SOD (superoxide dismutase), and CAT (catalase). PCA analysis displayed that cadmium severely affected rice plants, especially physiological indicators, but had little effect on T2 (P2 × H3) hybrids. Finally, the phenotypic data of the untreated hybrids and their parents were analyzed, and it was found that the number of seeds and yield per plant were the main contributions of the hybrids (Figure S9).

The root length of the P2 × H3 hybrids increased by 16.1% compared to before treatment, while the root length of the Cd-treated plants increased by 11.6%. There was no significant difference in root length between P2 and H3, and no significant difference in root length between P2-2x and H3-2x. When only the control or treatment groups were evaluated, there were no significant differences in root length growth between tetraploid and diploid hybrid (Fig. 5D). However, under cadmium stress, the root length of the P2 × H3 hybrid was reduced by only 28.0% compared to CK, with no significant difference. In contrast, the P2-2x × H3-2x hybrid dropped 33.7% compared to CK, with a significant difference. The shoot length of the P2 × H3 hybrid in the control group increased by 28.8%, whereas it increased by 16.4% under Cd exposure. The drop in shoot length was 43.0% compared to CK, which was much lower than that of the parents. The shoot length of the P2-2x × H3-2x hybrid was 13.8% in the control group and 8.6% in the Cd-treated group, and it reduced by 32.6%, which was likewise smaller than the parents (Fig. 5E). This indicates that the growth of the hybrid’s shoot length was more tolerant to Cd stress. The shoot length of the tetraploid hybrid (T2) was much greater than that of the diploid hybrid (D2), which indicates that the tetraploid hybrid exhibits greater Cd-induced stress tolerance than the diploid hybrid.

Rice root and shoot absorb Cd differently. The amount of Cd absorbed by the root is significantly higher than that of the shoot. The absorption of Cd by T2 shoot was 24.32% lower than that of D2. The absorption of cadmium by the root of T2 was 21.86% lower than that of D2 (Fig. 5F and G). Hybrids had significantly lower levels of cadmium in their roots and shoots than their parents, while the lowest levels of cadmium were absorbed in tetraploids. As for the parents, diploid and tetraploid parents’ growth significantly decreased under Cd stress, which might be attributed to Cd’s lower uptake in tetraploid plants than in diploid plants.

### Effects of Chlorophyll, Carotenoids, Metal Transporters, Non-Enzymatic, and Enzymatic Activity in Tetraploid Hybrids Under Cadmium Stress

There was no significant difference in chlorophyll a content between tetraploid and diploid under normal growth conditions. However, under Cd contamination, chlorophyll a was significantly reduced in all lines. Moreover, chlorophyll a’s content in diploid hybrids and their parents was significantly reduced compared to tetraploid hybrids and their parents (Figure S10). Under cadmium toxicity, tetraploid hybrids had higher chlorophyll a than their parents, and there was a non-significant difference between diploid hybrids and their parents. The contents of chlorophyll a decreased by 16.00% (1.63 mg g^− 1^ FW) under Cd toxicity compared to CK (1.37 mg g^− 1^ FW) in T2 hybrid. The contents of chlorophyll a decreased by 27.81% (1.17 mg g^− 1^ FW) under Cd toxicity compared to CK (1.62 mg g^− 1^ FW) in the D2 hybrid (Figure S10A). The contents of chlorophyll b decreased by 25.15% (0.51 mg g^− 1^ FW) under Cd toxicity compared to CK (0.68 mg g^− 1^ FW) in the T2 hybrid. The contents of chlorophyll b decreased by 28.84% (0.47 mg g^− 1^ FW) under Cd toxicity compared to CK (0.66 mg g^− 1^ FW) in the D2 hybrid. (Figure S10B). Under cadmium toxicity, the total chlorophyll content was significantly reduced (Figure S10C). The trend of carotenoids and chlorophyll b content was more consistent, and the carotenoid contents in the hybrids were higher. The carotenoid contents decreased by 33.31% (0.16 mg g^− 1^ FW) under Cd toxicity compared to CK (0.25 mg g^− 1^ FW) in the T2 hybrid. The contents of carotenoid decreased by 45.59% (0.13 mg g^− 1^ FW) under Cd toxicity compared to CK (0.24 mg g^− 1^ FW) in D2 hybrid (Figure S10D). Chlorophyll b and carotenoids were higher in hybrids than in parents. Under Cd toxicity, chlorophyll a, chlorophyll b, chlorophyll ab, and carotenoids decreased less in tetraploid hybrids than in diploids.

Hydrogen peroxide levels were significantly lower than parents in tetraploid and diploid hybrids. Under Cd toxicity, hydrogen peroxide content was significantly increased in all lines. The contents of hydrogen peroxide increased by 57.75% (92.31 nmol mg^− 1^ FW) under Cd toxicity compared to CK (58.52 nmol mg^− 1^ FW) in T2 hybrid. The contents of hydrogen peroxide increased by 90.68% (142.56 nmol mg^− 1^ FW) under Cd toxicity compared to CK (74.76 nmol mg^− 1^ FW) in D2 hybrid. Both hybrids’ hydrogen peroxide contents decreased significantly compared to their parent plants, whereas the H_2_O_2_ content remained nearly similar to that of the tetraploid progenitors in D2 (Figure S10E). Regarding changes in malondialdehyde content, there were non-significant differences between tetraploid and diploid hybrids and their parents under control treatment. There was no significant difference in MDA content in T2 and its parents under cadmium stress compared to CK. Under cadmium stress, the MDA content of D2 and its parents increased significantly compared with the CK (Figure S10F). Under cadmium toxicity, the line with the largest increase in malondialdehyde content was P2-2x, and the line with the smallest increase was T2 hybrid (Figure S10F). Relative to parents, glutathione was higher in T2 and D2 hybrids. Under cadmium toxicity, the content of GSH (Glutathione) decreased by 46.98% and 54.69% in tetraploid and diploid rice hybrids compared to their CK, respectively. The glutathione content decreased the least in the T2 hybrid, followed by the D2 hybrid (Figure S10G). T2 and D2 had significantly more CAT content than their parents, and T2 had significantly higher CAT than D2. T1 and D1 also have the same results. The contents of CAT decreased by 29.50% (8.93 U mgprot^− 1^ FW) under Cd toxicity compared to CK (12.66 U mgprot^− 1^ FW) in the T2 hybrid. Diploid parents decreased the most, with paternal and maternal decreases of 56.90% and 59.47%, respectively (Figure S10H).

In normal environments, the SOD activity of tetraploid hybrids (T2) was not significantly different from that of parents, and diploid hybrids (D2) were significantly higher than those of their parents (H3-2x). Under cadmium toxicity, the content of SOD in tetraploid hybrids decreased by 22.88. Under cadmium toxicity, the SOD content in diploid hybrids (D2) decreased by 39.83%. The proportion of tetraploid declines is less than that of diploids (Figure S10I). There was no significant difference in POD (peroxidase) content between tetraploid hybrids (T2) and diploid hybrids (D2) and their parents under control conditions, but POD content decreased under cadmium toxicity. The POD content in tetraploid hybrids decreased by 28.74%, with the smallest decline, followed by diploid hybrids, which decreased by 41.67%. The parents of the two hybrids decreased the most, and there was no significant difference in POD content in the parents under Cd toxicity (Figure S10J). The physiological changes under salt stress were consistent with those under Cd stress (Figure S11).

In addition, we verified the expression changes of several genes on cadmium tolerance in neo-tetraploids and the diploids that reverted from them, autotetraploid and their diploids. It was found that the expression levels of *OsABCC1*,* OsHMA3*, and *OsNRAMP1* in the roots of neo-tetraploid rice were increased under cadmium stress, which was higher than that of diploid (Figure S12). Primers for qPCR genes are shown in the supplementary table (Table S6). These results indicate that proteins such as ABCC transport cadmium into vacuoles for detoxification (Song et al. 2014). However, the expression levels of *OsABCC1*, *OsHMA3*, and *OsNRAMP1* decreased in autotetraploid rice under cadmium stress. Autotetraploid rice may lack the strong detoxifying capability characteristic of neo-tetraploid rice. The expression of *OsERF1* decreased in the neo-tetraploid root under cadmium stress and decreased more in the neo-tetraploid rice. The expression of *OsERF1* in leaves under cadmium stress was opposite to that in roots, which may be due to the fact that *OsERF1* mainly responds to cadmium stress in leaves. *OsERF1* was also highly expressed in the leaves of hybrids with neo-tetraploid parents (Figure S12). However, the expression of *OsNAC9* under cadmium toxicity was increased in the neo-tetraploid and it’s diploid. The expression of these two transcription factors, which are closely related to abiotic stresses, was different in tetraploid and diploid rice, which might explain that *OsNAC9* is a little more responsive to the material used in this experiment (Huang et al. 2021; Redillas et al. 2012). Moreover, there were notable differences in the expression levels of *OsNRAMP1*, *OsABCC1*, and *OsHMA3* between plants exposed to Cd and those untreated. In response to Cd toxicity, there was a marked increase in the expression of *OsNRAMP1*, *OsABCC1*, and *OsHMA3*. Typically, tetraploid parents and hybrids showed differential expressions when exposed to Cd toxicity compared to their diploid counterparts. Consistent with morphological and biochemical evidence, salt-related resistance genes like *OsARF18* and *OsIAA18* also showed distinct expression patterns across diploid and tetraploid rice hybrids.

### Tolerance Difference Between P2-2x × H3-2x and P2 × H3 Hybrids

**Fig. 6:**
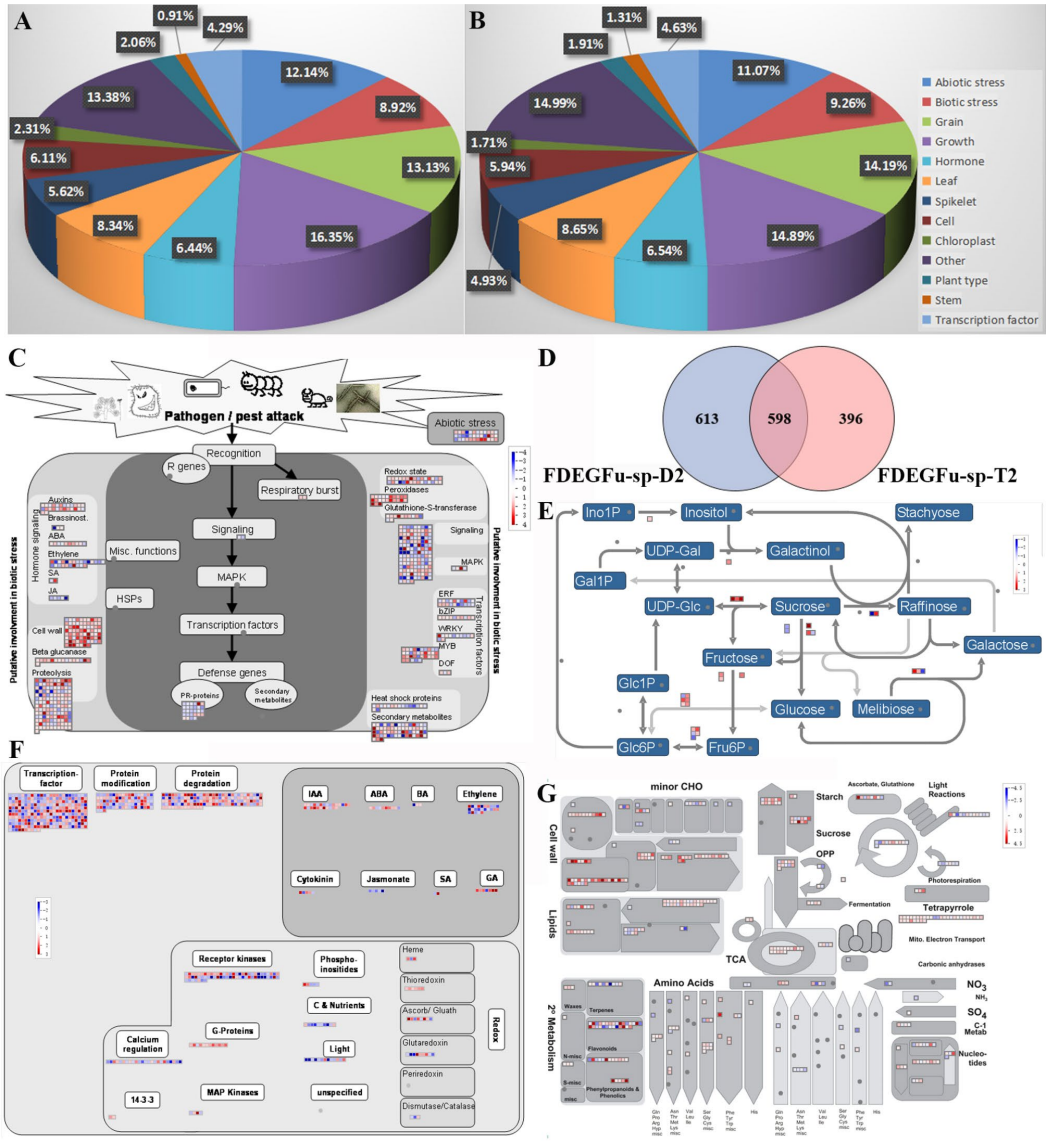
DEGFu-sp-D2 and DEGFu-sp-T2 gene proportion and enrichment pathways related to each trait. (**A**) Functional difference between D2 and its parents; (**B**) Functional classification of gene set with the functional difference between T2 and its parents; (**C**) Biological stress pathway of D2 vs. T2; (**D**) Venn diagram of FDEGFu-sp-D2 and FDEGFu-sp-T2; (**E**) Raffinose metabolism process of D2 vs. T2; (**F**) Participate in the redox process of D2 vs. T2; (**G**) Overview of metabolic pathways of D2 vs. T2. D2 is a diploid hybrid of P2-2x and H3-2x; T2 is a tetraploid hybrid of P2 and H3

A total of 1211 and 994 genes with known functions that differ between D2 and T2 and their respective parents were identified, with 598 genes being common (Fig. 6D). This suggests that roughly half of the parental genes in the tetraploid doubling persist from the diploid cross. The percentage of DEGFu-sp-D2 genes related to plant development was 16.35%, followed by 13.13% for rice grains, 12.14% for abiotic stress, 8.92% for biological stress, 6.44% for plant hormones, and 8.34% for leaves (Fig. 6A). In DEGFu-sp-T2, the proportion of genes related to plant development, grain, abiotic stress, biological stress, plant hormones, and leaves is 14.89%, 14.19%, 11.07%, 9.26%, 6.54%, and 8.65%, respectively (Fig. 6B). The differential gene proportions in D2 and T2 hybrids were almost identical, but diploid hybrids have more differences than tetraploid hybrids. Nevertheless, tetraploid hybrids have more grain-related genes, significantly increasing their 1000-grain weight compared to their parents.

DEG analysis comparing D2 (diploid) and T2 (tetraploid) hybrids showed gene changes related to protein hydrolysis and signal pathways. More than 20 genes related to peroxidase and auxin, and more than 70 genes related to cell wall, were up-regulated, indicating cell protection (Fig. 6C). There was also significant activity in cell wall metabolism, particularly in cell wall protein, pectin esterase, cell wall modification, cellulose synthesis, pectin lyase, and polygalacturonase (Fig. 6G). Up-regulation of genes related to sucrose, starch synthesis, degradation, and raffinose metabolism involving sucrose was observed (Fig. 6E). Raffinose metabolism is important in plant stress resistance, suggesting tetraploid hybrids may have stronger stress resistance than diploid ones. Transcription factors, along with protein modification and degradation, played a crucial role at the regulatory level. The corresponding signal links were receptor kinase, calmodulin, and G protein, which complemented each other (Fig. 6F).

## Discussion

### Tetraploid Hybrid Rice Displays Dominance Through the Coordination of Multiple Development Processes Dominated by Sugar Metabolism

Breeding excellent germplasm relies on heredity and variation, where rice polyploidy increases the genome, preserves genetic information, and leads to more recombination mutations and new traits, thus providing more resources for crop breeding. Heterosis, or hybrid vigor, is mainly due to metabolic pathways, such as galactose metabolism in maize hybrids (Dan et al. 2020) and stronger photosynthetic efficiency and sugar metabolism in triploid loquat hybrids (Wang et al. 2022).

Transcriptome analysis of leaf tissue from tetraploid and diploid hybrids showed non-significant differences, while the primary biochemical process occurs in spikelets at the heading stage. Metabolism becomes stable five days after fertilization, with sugar and starch metabolism and cell wall expansion in the spikelet leading to larger grain size in tetraploid hybrids. *Indica* × *japonica* and *indica* × *indica* tetraploid hybrids showed an increased 1000-grain weight of 16.05 g and 12.3 g, respectively, compared to diploid hybrids. Tetraploid rice has larger biomass and grains but is limited by seed setting rate. However, using tetraploid rice with a high seed setting rate as a germplasm resource can produce hybrids with obvious super parent advantages. In particular, H3 can be used as a widely assorted germplasm to contribute to heterosis. Metabolic analysis showed that T2 has a more vigorous sugar metabolism, leading to more abundant grain starch than D2 (Figure S13). Grain development is heavily influenced by metabolism, but the combination of hormone signal transduction, environmental response mechanisms, regulation of transcription factors, and protein modifications throughout the entire growth cycle ultimately leads to heterosis. In addition, an inhibitor, *OsSGT1*, interfered with rice immunity and decreased its expression in D2 hybrids (Chen et al. 2022). *OsBLE2* (*Os07g0650600*) is a brassinolide-enhancing gene that regulates the growth and development of rice (Yang et al. 2003). *OsINO1* (*Os03g0192700*) can synthesize phytic acid, and its expression is elevated in D2 hybrids (Perera et al., 2019). *OsAlaAT1* (*Os10g0390500*) is an alanine transferase that regulates rice endosperm starch synthesis and can regulate rice nitrogen utilization (Yang et al. 2015; Fang et al. 2022). The combined action of these genes may be a crucial factor in heterosis.

However, some scholars argue that heterosis alone cannot account for the superior performance of hybrids and that it is only revealed when comparing the weaknesses of homozygotes. Currently, there are 15 genes associated with heterosis, including *Os01g0274800* (*CSA*), *Os06g0610300* (Conserved hypothetical protein), *Os01g0917500* (*MSP1*), *Os04g0208600* (Cyclin-like F-box domain containing protein), *Os07g0624900* (*SKP1*), *Os12g0298600* (*OsEAF6*, *HWE1*), *Os06g0157700* (*Hd3a*), *Os09g0529300* (*TAC1*), *Os08g0174500* (*Ghd8*), *Os01g0883800* (*sd-1*), *Os04g0615000* (*NAL1*), *Os06g0275000* (*Hd1*), *Os06g0650300* (*GW6a*), *Os08g0509600* (*IPA1*), and *Os09g0441900* (*DEP1*). In this experiment, only the *OsEAF6* and *SKP1* genes showed a significant increase in expression in the hybrid combinations compared to their parents. In contrast, other heterosis-related genes did not show significant changes, and some even exhibited a significant decrease in expression in hybrids. This suggests that the increase in cell wall, cell division number, and the linkage of the entire metabolome, rather than a single major gene, may be responsible for this phenomenon. Moreover, the ability to resist environmental stress throughout the entire growth cycle ultimately leads to increased yield.

### H3 can Provide Germplasm Resources to Enhance Abiotic Stress Resistance and Overcome *Indica* × *Japonica* Hybrid Sterility

Under cadmium toxicity and salt stress, the root length of H3 consistently exceeded that of the other parent. Hybrids exhibit larger root lengths, potentially attributable to the genetic composition conferred by H3. For example, the germination rate under salt stress was higher, which was similar to that of tetraploid hybrids. It had lower MDA and H_2_O_2_ contents, and higher GSH and POD contents under salt stress. Given the elevated ROS, H3 may be more effectively resolved high oxidative stress. In addition, the expression of *OsARF18* negatively regulated salt tolerance gene was almost not expressed in H3. Under cadmium toxicity, it also has a high antioxidant content of superoxide dismutase and non-enzymatic antioxidants. In conclusion, it is indicated that the neo-tetraploid rice H3 has high resistance to abiotic stress.

The infertility of hybrids resulting from the incompatibility between *indica* and *japonica* genomes remains a significant concern for researchers. Genetic sterility of distant crosses limits the efficient use of *indica*-*japonica* hybrids. Therefore, there is a need for a way to overcome the low seed setting rate. The hybrids employed in this investigation also exhibited a high level of expression of the genes associated with heterosis that have been reported. For example, genes such as *Hd1*, *Hd3a*, *sd-1*, and *OsEAF6* were highly expressed in the hybrids. However, not all reported heterosis-related genes were highly expressed in hybrids. The different material backgrounds may result in redundant functionalities of certain genes across different environments. One approach is to create single-segment substitution lines through *indica* × *japonica* hybrids (Zhang 2022) or identify cultivars with neutral genes (Shahid et al. 2013), which can yield valuable genetic resources with diverse genetic characteristics, thus providing a good source of germplasm for hybrid rice breeding. The *Sa* locus is responsible for male hybrid sterility but can be overcome by knocking out or silencing the *SaF/SaM* allele (Xie et al. 2017). The increase in pollen sterility rate in tetraploid was due to enhanced epistatic interactions between pollen sterility loci, thereby changing the expression profiles of important meiosis-related or meiotic stage-specific genes (Wu et al. 2015a). Here, the pollen fertility of the hybrid and its parent was normal. This may be due to H3 and H3-2x carrying neutral genes that increase pollen fertility (Chen et al. 2020). According to Qian et al. (2016) (Qian et al. 2016), the heterosis between subspecies (*indica* × *japonica*) is stronger than that within subspecies due to greater genetic diversity. While there is some reproductive isolation between *indica* × *japonica* hybrid rice, intermediate types of *indica* × *japonica* hybrid rice can overcome sterility (Ikehashi and Araki 1984). S5 allele system was identified as the primary factor regulating reproductive disorders in intersubspecific hybrids (Chen et al. 2008). The seed setting rate of the T2 hybrid can reach 74.48%, and that of D2 was 40.60%. After chromosome doubling, the autotetraploid hybrid overcame the reproductive isolation between *indica* × *japonica* rice.

### Tetraploid Hybrids Exhibited Strong Tolerance to Abiotic Stress

**Fig. 7:**
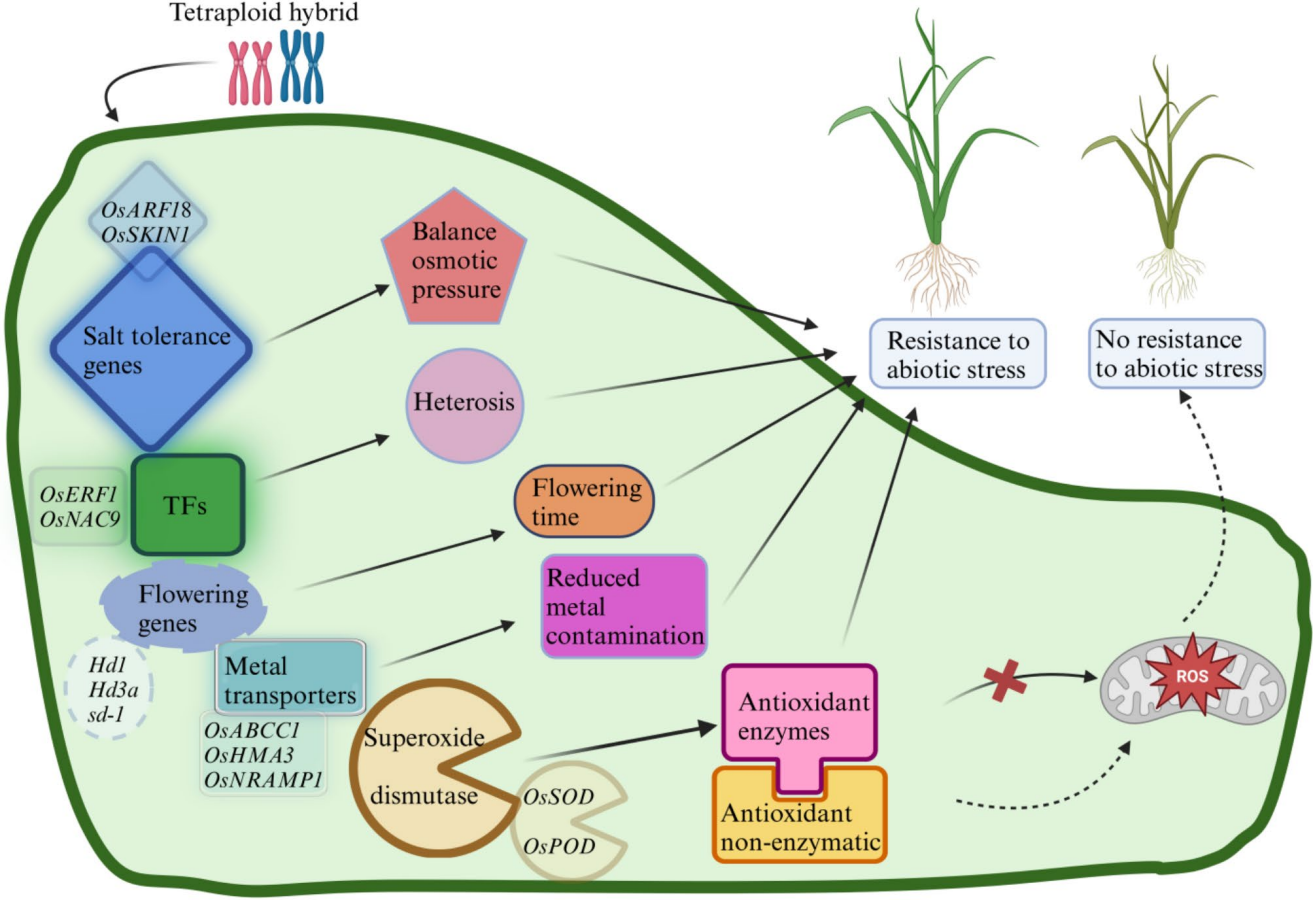
Mechanism of abiotic stress resistance in tetraploid rice hybrids. The mechanisms encompass essential resistance and metal transporters and defensive responses: *OsABCC1* (ATP-Binding Cassette transporter family in rice), *OsHMA3*, *OsNRAMP1* (Oryza sativa Natural Resistance-Associated Macrophage Protein 1), *OsARF18*, *OsNAC9* (resistance genes), transcription factors, reactive oxygen species (ROS), and the antioxidant defense system. Root and shoot Cd deposition in rice plants is heightened due to reduced chlorophyll levels, oxidative stress, and inhibited growth resulting from cadmium toxicity. However, the detrimental effects of Cd exposure were mitigated by polyploid rice hybrids, which reduced Cd accumulation in roots and shoots, enhanced chlorophyll concentrations, and strengthened the plant’s antioxidant defense system

In this experiment, tetraploid hybrids demonstrated greater salt tolerance and resistance to cadmium toxicity (Fig. 7). We have proposed a genetic mechanism of high tolerance in hybrids compared to parents in diploid and tetraploid rice. Heterosis produced by tetraploid hybrids causes changes in genes such as transcription factors, flowering genes, salt stress-related genes, metal transporters, and superoxide dismutase. The change in transcription factor expression had a promoting effect on the growth and development of rice. The synergistic effect of flowering genes allows hybrids to bloom and fertilize at a more suitable time and improves the seed setting rate of tetraploids. Metal transporters, salt stress-related genes, and superoxide dismutase can regulate the activities of some enzymes and non-enzymes and better scavenge reactive oxygen species. Consequently, the co-regulation of several factors under abiotic stress enhances the tolerance of tetraploid hybrids.

During the growth process of the microspore pollen stage, heading stage, and 5 days after fertilization, the four hybrids display greater tolerance to biotic and abiotic stresses compared to their parents. Additionally, the doubled tetraploid hybrid demonstrates a more active metabolic process. The transcription levels of tetraploid and diploid hybrids and their parents can be seen in the additional file (Table S7). Many up-regulated gene expressions regarding environmental responses in D2 and T2 hybrids existed. *OsSKIN1* (*Os09g0499000*) is a negative regulator of *snrk1a* interaction, which regulates the source-pool signaling process under abiotic stress at the seedling stage and is highly expressed under drought stress (Lin et al. 2014). RNA-Seq results were consistent with our morphological, biochemical, and physiological data under Cd and salinity stress, which showed high resistance in tetraploid hybrids compared to parents and diploid hybrids. *OsARF18* is a negative regulator of glufosinate ammonium resistance and salt tolerance (Xia et al. 2023). Under salt stress, *OsARF18* was highly expressed in D2 and its parents, but almost no expression in T2 and its parents (Figure S11). However, other positively regulated salt tolerance genes, such as *OsIAA18* (Li et al. 2020; Wang et al. 2021), also showed differential expression patterns between D2 and T2 hybrids (Figure S11). This may result from the variable functional intensity of several salt tolerance genes during saline stress. Alternatively, there exists a hierarchical arrangement for genes that function in different environments. Here, D1, T1, D2, and T2 showed certain production advantages. Analysis of agronomic traits during the early and late seasons indicated that the early season of hybrids outperformed the late season. However, tetraploid hybrids fluctuated less between the early and late seasons than diploid hybrids (Figure S14), indicating a greater tolerance to temperature changes. This aligns with the observation that the environmental impact of *indica* and *japonica* hybrid rice weakens with decreasing latitude (Xu et al. 2019).

### Under Cadmium Toxicity and Salinity Stress, Changes in Chlorophyll, Metal Transporters, Non-Enzymatic, and Enzymatic Activity in Hybrids were More Stable than in Parents

The decrease in tetraploid hybrids was the least, indicating that salinity and Cd toxicity had less effect on tetraploid hybrids. Tetraploids exhibit greater tolerance under abiotic stress, such as autotetraploid rice was more tolerant than diploid under salt, alkali, and saline stress (Wang et al. 2023). Under abiotic stress, excessive superoxide radicals are produced, which increases the content of hydrogen peroxide and MDA (Karam et al. 2016). The levels of H_2_O_2_ and MDA in both diploid and tetraploid rice were increased under cadmium stress. The increase of peroxidase activity in tetraploid under cadmium toxicity converts reactive oxygen species into non-toxic substances and maintains cell homeostasis. Tetraploid rice also has a stronger defense system under arsenic stress (Ghouri et al. 2023b). Similarly, under the stress of cadmium and salt, the activities of antioxidant enzymes, such as SOD and POD, were significantly increased in T2 hybrid. In this study, the expression levels of *OsABCC1* and *OsHMA3* genes were increased under cadmium toxicity. Significant disparities existed in the expression levels of metal transporters between tetraploid hybrids and their diploid counterparts and progenitors. Chelates with heavy metals may remain in vacuoles without transportation, hence conferring significant resistance to cadmium (Hayashi et al. 2017), or it may also be by changing the root structure, such as stomatal size, to improve tolerance (Redillas et al. 2012). In this study, we found two genes, *HRAN-1* (*Os03g0823400*) and *HRAN-2* (*Os01g0314800*), in diploid and tetraploid rice, which corroborate our prior research on cadmium treatment under hydroponic conditions (Ghouri et al. 2023a). The expression levels of *HRAN-1* and *HRAN-2* were significantly higher in tetraploid and cadmium-treated roots.

Environmental stress causes changes in the chlorophyll content of crops, which affect the photosynthetic rate, and the chlorophyll content of hybrids and inbred lines differ under environmental stress (Avramova et al. 2016). In this experiment, the higher chlorophyll and carotenoid contents in the hybrids could reduce the decline of rice photosynthesis capacity and accumulate sufficient “sources”. Under salinity and cadmium toxicity, the activity of antioxidant enzymes decreased less in hybrids. This is the stress mechanism of rice under environmental stress, which scavenges free radicals through the increased activity of antioxidant enzymes (Cao et al. 2019). However, the hybrids in this experiment retained more antioxidant enzyme activity under environmental stress and strongly resisted oxidative damage in rice. Hybrids have significantly higher levels of GSH than their parents, and GSH acts as a reducing agent to reduce arsenate to As(III) by arsenic acid reductase (Bhat et al. 2021). Tetraploid is more resistant to Cd than diploid and is responsible for cellular homeostasis, plant tissue structure, dynamic physiological and gene expression changes and other aspects. Therefore, hybridization with H3 as a germplasm resource may lead to more varieties with strong environmental tolerance.

## Conclusions

Polyploid rice had strong heterosis in many traits, such as number of grains, yield per plant, and biomass. Transcriptome analysis revealed that most of the genes were non-additive. In addition, there were significant changes in the expression of some TFs and genes in response to the environment. Most of these genes were associated with biotic and abiotic stresses. The study investigates the effects of hybridization and polyploidy on different morphological metrics, RNA-seq, and physiological indicators in rice under Cd and salt stress. In summary, some genes of ERF, bZIP, WRKY, MYB, and DOF transcription factor families have significant changes, such as *OsNAC19*, *OsERF1*, *OsWRKY21*, and *OsSKIN1*, which are involved in environmental response. In addition, some genes that were up-regulated in hybrids have also been shown to be related to environmental responses. Under cadmium toxicity, the expression of metal transporters (*OsABCC1*,* OsHMA3*, and *OsNRAMP1*) and TFs (*OsERF1* and *OsNAC9*) in tetraploid rice was significantly different than that of diploid rice. Hybrids exhibited more tolerance to Cd and salinity than their parents, with tetraploid hybrids demonstrating even higher tolerance than diploid hybrids. Tetraploid hybrids had the highest chlorophyll content, the most stable antioxidant enzymes, and a low level of ROS under heavy metal toxicity. Therefore, tetraploid hybridization may be a natural resource for improving rice resistance. The present research establishes a foundation for a more thorough comprehension of how hybridization can improve plant resistance to environmental stress factors. Although there are certain limits, our findings provide significant insights for future research endeavors.

## Materials and Methods

### Plant Materials

The materials were planted at South China Agricultural University’s research facility in Guangzhou, Guangdong Province. The diploid materials were designated as H3-2x (*indica*), P1-2x (*indica*), and P2-2x (*japonica*), while the corresponding one autotetraploid materials were referred to as H3 (*indica*), P1 (*indica*), and P2 (*japonica*), respectively. The current investigation utilized two diploid hybrids, D1 (P1-2x × H3-2x) and D2 (P2-2x × H3-2x), as well as two tetraploid hybrids, T1 (P1 × H3) and T2 (P2 × H3) (Figure S15). In our laboratory, tetraploid rice was obtained by doubling colchicine. H3 is a high-fertility tetraploid rice bred by crossing two tetraploids and selecting them after multiple generations(Guo et al. 2017). H3 is a high-fertility autotetraploid rice bred by our research group, and H3-2x is a diploid rice recovered from H3. P1 or P2 means parents; D1 or D2 means diploid hybrids; T1 or T2 means tetraploid hybrids. All materials were planted in three replications. For the salt stress treatment, 30 seeds were placed in each dish, with four replicates. The duration of daylight and darkness was equal, lasting 12 h, while the temperature was 30 °C. The seeds were immersed in a solution containing a concentration of 200 mM NaCl. On the first day, each dish received an additional 20 ml of NaCl, followed by a daily addition of 10 ml solution. The same volume of water was used as a control. The germination rate is observed at the same time every day. The seeds were soaked for three days for the cadmium treatment experiment and then transferred to the plastic pots after germination. At first, seedlings were treated with 1/2 rice nutrient solution, then full-strength nutrient solution. It was treated with 100 µM cadmium chloride two weeks later for 15 d.

### Evaluation of Agronomic Traits and Heterosis Analysis

WPS 2022 and IBM SPSS Statistics 25.0 were used for data analysis and processing. Root length and shoot length were measured after Cd stress. The heterosis indicators evaluated in this article include mid-parent advantage (MPH), over-parent advantage (HPH), and over-standard parent advantage (SPH). Take 20 plants from each material to measure the statistics of each character. The following formulas were used to calculate heterosis: MPH = (F_1_-MP)/MP × 100%, HPH = (F_1_-HP)/HP × 100%, SPH = (F_1_-SP)/SP × 100%. Fifteen plants were selected for each strain to be investigated, and the average value of the early and late seasons was finally displayed.

### Determination of Cd Contents

The determination of cadmium content was determined by HNO_3_-HClO_4_ digestion by digesting plant samples and then determined by atomic absorption spectrophotometry. The specific method refers to the previous research in this experiment (Lai et al. 2023). Each experiment has three replicates.

### Determination of Chlorophyll a, Chlorophyll b, and Carotenoids

Take 0.5 g of fresh leaves after ten days of treatment and soak them in 95% alcohol for 48 h. Take 200 µL of the supernatant and assay under a microplate reader (UV-1700; Shimadzu, Japan). The absorption values of chlorophyll a, b, and carotenoids were 645 nm, 479 nm, and 663 nm, respectively.

### Malondialdehyde and H_2_O_2_

The detection of MDA was based on the Heath and Packer methodology (Heath and Packer 1968). The specific method refers to the previous research in this experiment (Ghouri et al. 2023a; Lai et al. 2023). The supernatant was mixed with 1 mL of 10 mM kH₂PO₄ buffer (500 µL) and 1 mL of 1 M KI, and absorbance was detected at 390 nm.

### Estimation of GSH, SOD, POD, and CAT

A certain amount of leaves was digested in 50 mM Na₃PO₄ solution (v/v) (pH 7.8) and 13,000 rpm in a centrifuge at 4 °C for 15 min. The supernatants of GSH, SOD, POD, and CAT were to be measured. GSH is determined using the A006-2-1′ kit from Nanjing Jiancheng Biotechnology (Lai et al. 2023). SOD, POD, and CAT were determined using the corresponding SOD (WST-1 method, A001-3-2), POD (Visible light method, A084-3-1), and CAT (Visible light method, A007-1-1) kits, respectively. Each experiment is performed three times with three replicates per sample. All the above kits were purchased from Nanjing Jiancheng Biological Company (www.njjcbio.com*).*

### Statistical Analysis of PCA, Correlation, and Effect Size

PCA reduces multi-dimensional data to two-dimensional data so that it can easily analyze the contribution of a principal component to the target phenotype. The PCA is calculated using the theory of maximum variance, plotted using a web https://www.bioinformatics.com.cn, and the ellipse has a confidence interval of 68. Effect sizes were computed using partial Eta home remedies and were shown as *Cohend’f* results. The results of *Cohend’f* were calculated using SPSSAU software. The correlation coefficient was calculated using the Pearson formula, and the results were plotted using the website (https://www.omicstudio.cn/home).

### Analysis of Pollen and Embryo Sac Fertility

I_2_-KI (1%) solution was used to stain anthers extracted from panicles (Ghouri et al. 2019). The anthers were excised and positioned on a glass slide. One to two drops of the dye solution were applied, and the anthers were crushed using tweezers. After two minutes, the tissue was extracted, and the cover was positioned on the microscope for examination. Five plants were selected for each line, three branches were selected for each plant, and three spikelets were selected for iodine staining for each branch. The stained anthers were observed under a Motic BA200 optical microscope at a 10 × magnification. Five representative visual fields were captured using Motic Images Advance 3.2 software for each slide, and a ruler was added to the subsequent images using Adobe Photoshop software. The pollen fertility was calculated based on the type of abortive pollen grains.

Twenty plants were sampled from each line, and 200 spikelets with yellow anthers and middle and upper parts were taken. To prepare the ovary, young panicles were soaked in FAA solution (50% ethanol: glacial acetic acid: formaldehyde = 89:6:5) for 24 h. The specific method refers to the previous research in this experiment (Zhao et al. 2024).

### RNA Extraction, cDNA Library, and RNA-Seq

To perform RNA-seq, samples were collected from flag leaves at the microspore stage(Table S8), rice heading stage, and 5 days after fertilization, as well as spikelets at the rice heading stage and 5 days after fertilization (without anthers). The samples were immediately frozen in liquid nitrogen for 2 min and then stored at -80 °C for RNA extraction using the TRlzol (AG RNAex Pro Reagent) method. The extracted RNA was used to prepare a cDNA library. After library inspection, the libraries were pooled according to the target offline data volume and sequenced using the Illumina platform. Data was filtered and compared with the Nipponbare genome (Oryza_sativa. IRGSP_1.0). Differential genes were detected using DESeq2_ EdgeR, with genes having FDR significance < 0.01 and absolute multiple change value ≥ 2 selected for further analysis. Enrichment analysis of differential genes was performed using AgriGO and KEGG databases.

### DEGs, GO, and KEGG Analysis

To detect differential genes, we utilized DESeq2 and EdgeR, filtering for genes with FDR significance < 0.01 and log2FC ≥ 2 for subsequent analysis. Next, we employed AgriGO (http://bioinfo.cau.edu.cn/agriGO/) and the Gene Ontology database to enrich and analyze the differential gene set, followed by clustering analysis using specialized software. To perform metabolic enrichment analysis of the differential genes, we utilized the KEGG database (Kyoto Encyclopedia of Genes and Genomes) and drew a map using MapMan and TBtools1.1.

### qRT-PCR

In order to verify the accuracy of the transcriptome, we selected 30 genes based on the expression in spikelet tissues for qPCR analysis. The qPCR quantitative enzyme was amplified using the Yeasen reagent kit HB210720 via a two-step method with a procedure of 95 °C for 5 s and 60 °C for 30 s. Ubiquitin was used as the internal reference gene. We calculated the relative expression levels using the formula F = 2^^−ΔΔCt^ (Livak and Schmittgen 2001).

## Electronic Supplementary Material

Below is the link to the electronic supplementary material.

Supplementary Material 1

Supplementary Material 2

## Data Availability

The raw reads of RNA-seq were deposited in the NGDC BIG submission with accession ID PRJCA037357.
