# Supplementary material for: Interspecific Hybridization Enhanced Tolerance to Salinity and Cadmium Stress Through Modifying Biochemical, Physiological, and Resistance Gene Levels, Especially in Polyploid Rice: A Sustainable Way for Stress-Resilient Rice
Source: Rice (N Y). 2025 Mar 22;18:19. doi: 10.1186/s12284-025-00776-6 (PMC11928717; doi:10.1186/s12284-025-00776-6)
Supplement: Supplementary file 2 — Supplementary Material 2 [file 12284_2025_776_MOESM2_ESM.docx]

**Supplementary Figures**


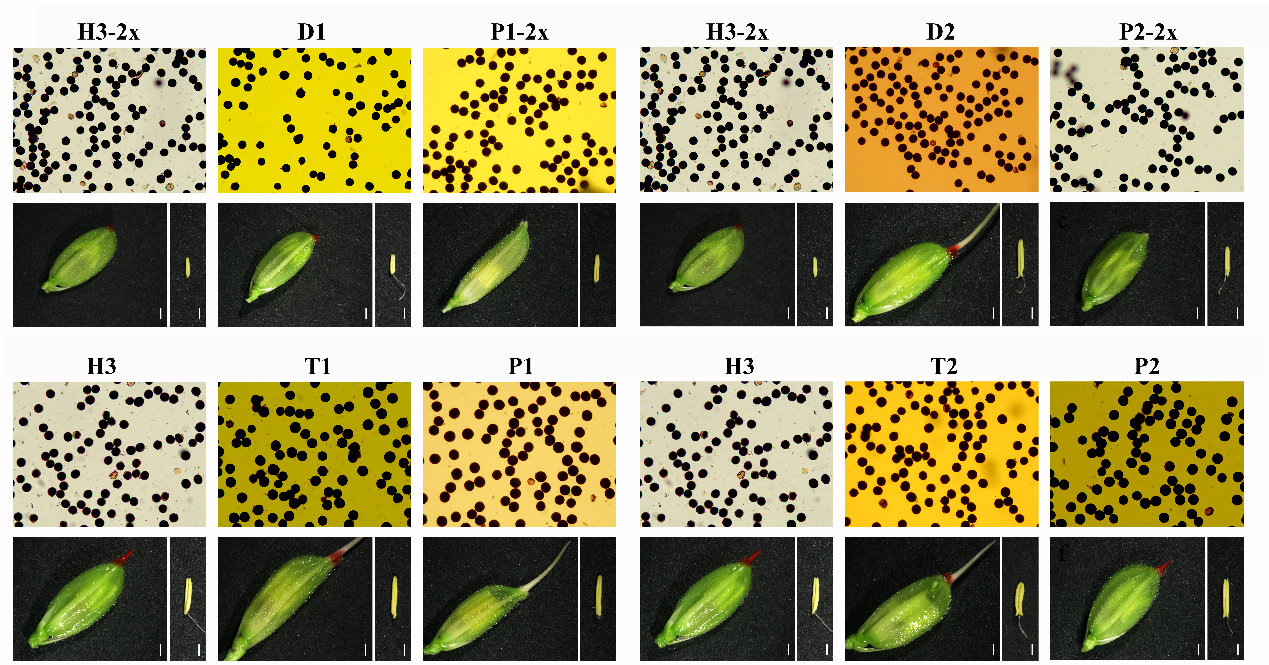


Figure S1 Anther morphology and pollen fertility evaluation by I_2_-KI. Scale bar: 100 μ m

D1 is a diploid hybrid of P1-2x and H3-2x; T1 is a tetraploid hybrid of P1 and H3; D2 is a diploid hybrid of P2-2x and H3-2x; T2 is a tetraploid hybrid of P2 and H3.


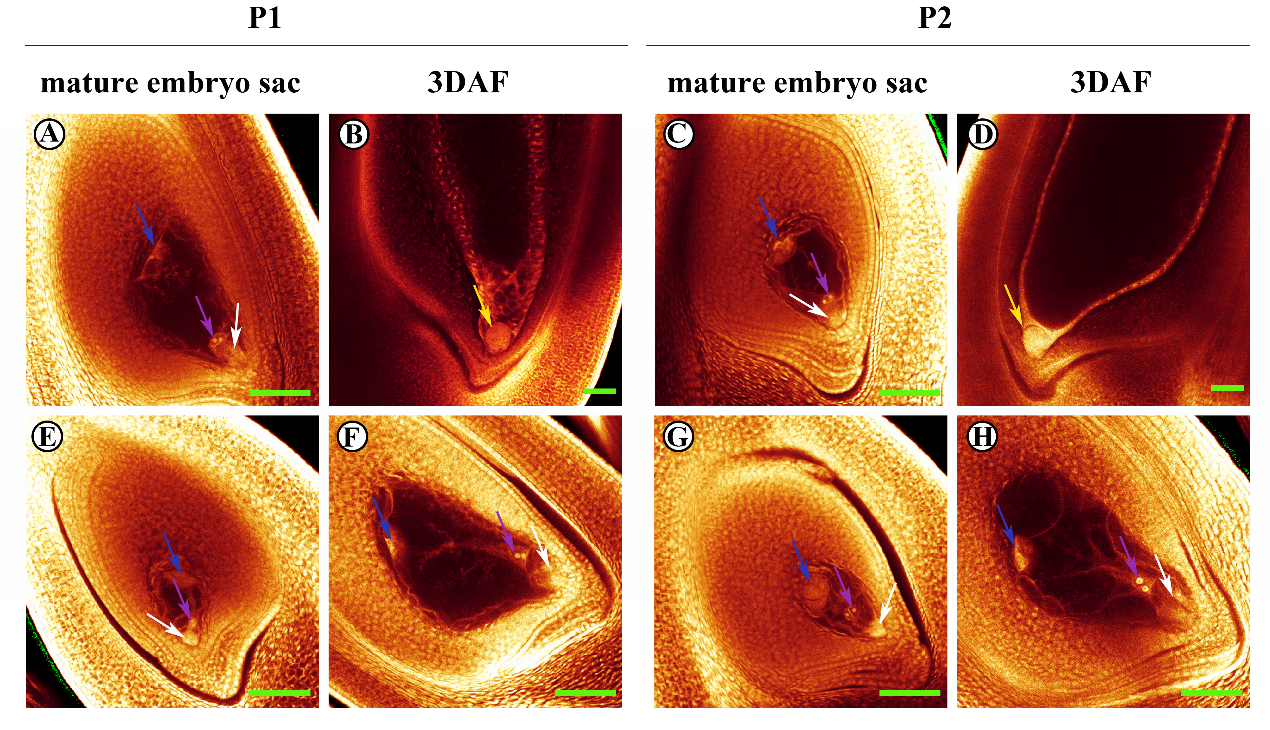


Figure S2 Observation of embryo sac of P1 and P2 at maturity and 3 days after fertilization.

(A, E) Normal development of embryo sac of P1 at maturity; (B) The normal development of embryo sac of P1 material 3 days after fertilization; (C, G) Normal development of embryo sac of P2 at maturity; (D) The normal development of embryo sac of P2 3 days after fertilization; (F) The embryo sac of P1 material was not fertilized normally 3 days after fertilization; (H) The embryo sac of P1 was not fertilized normally 3 days after fertilization. The blue arrows indicate antipodal cells, The purple arrows indicate polar nuclei, and The white and yellow arrows represent egg cells and globular proembryo, respectively. Scale bar: 40 μ m.


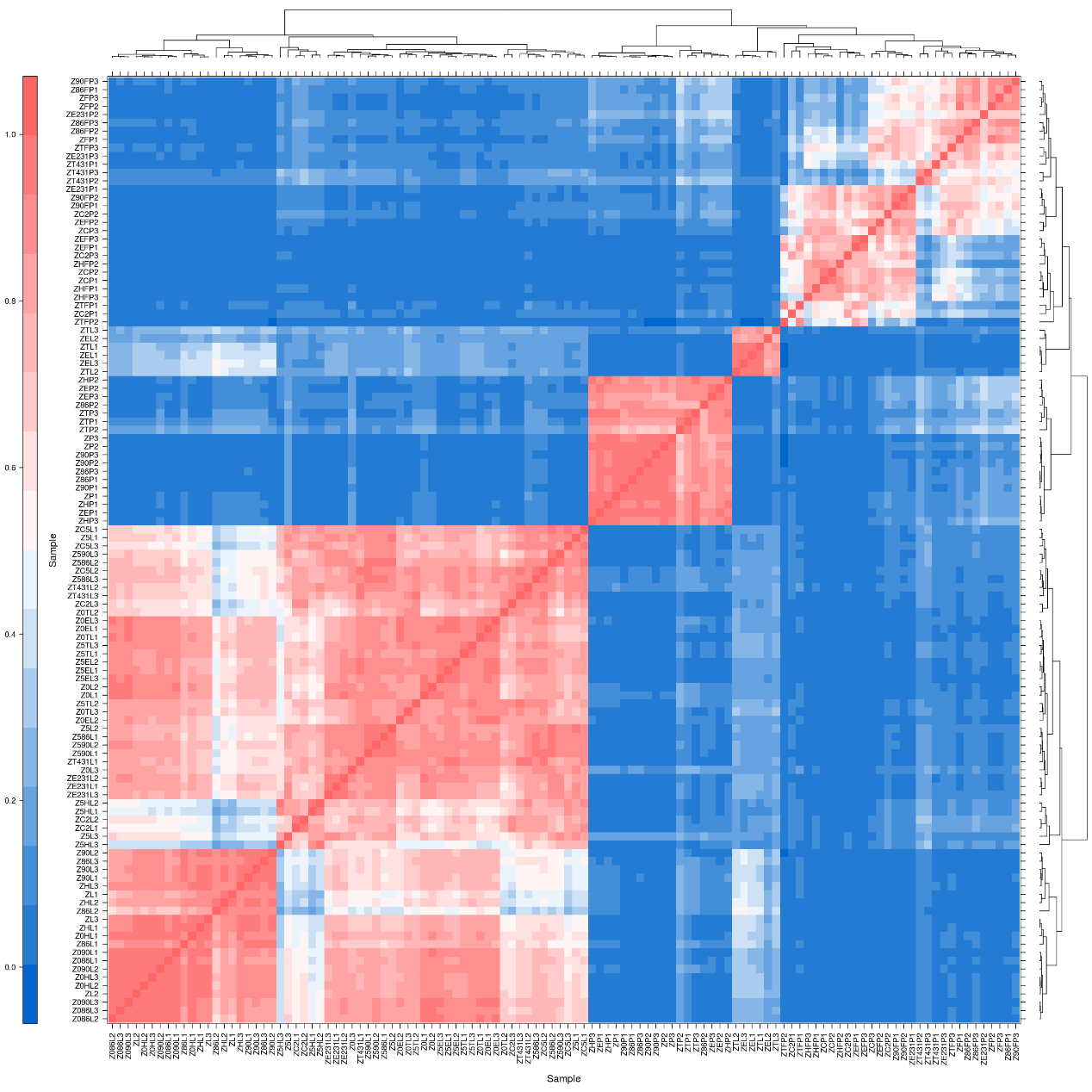


Figure S3 Correlation analysis between different samples.


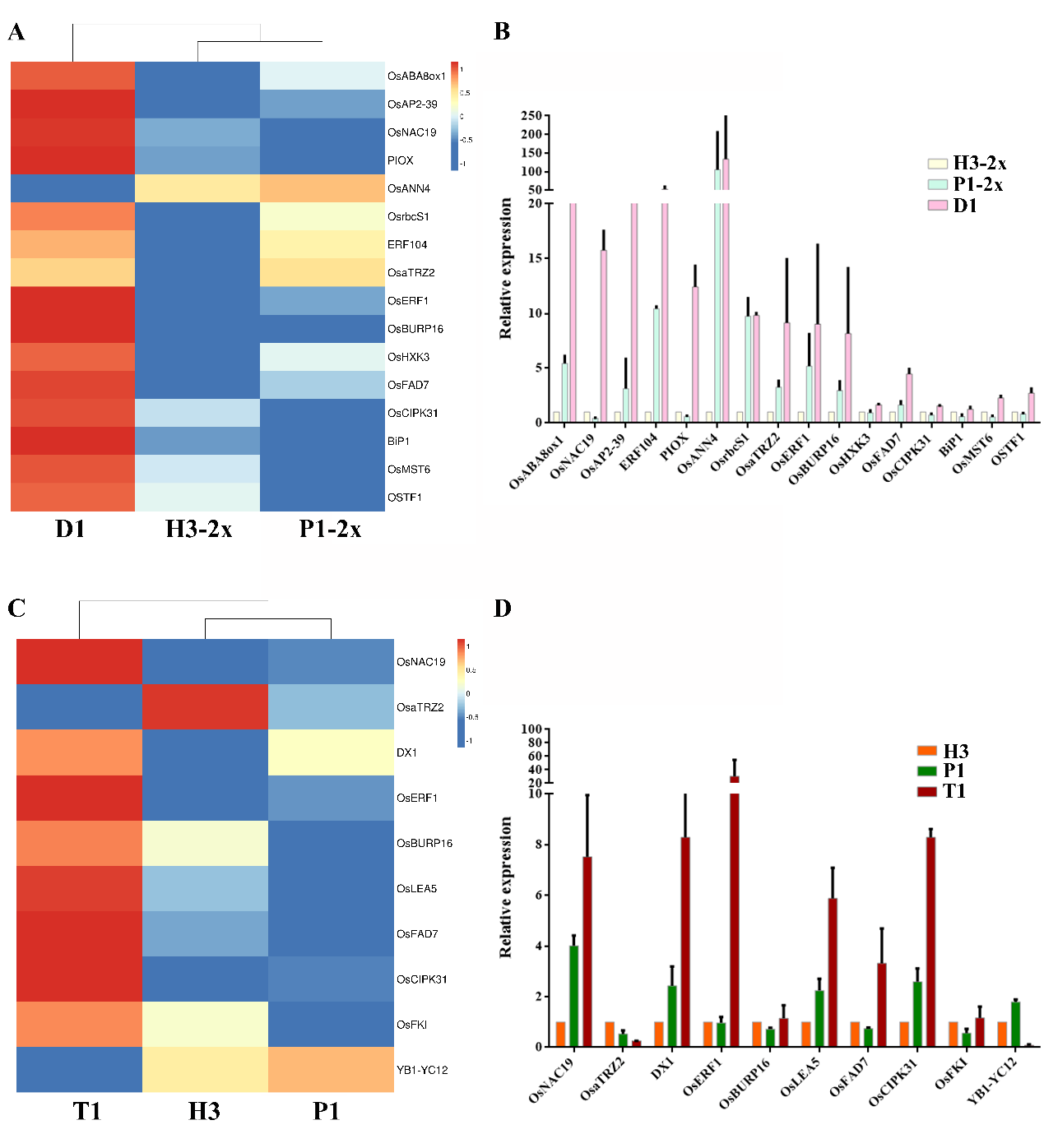


Figure S4 Transcriptome analysis and qPCR verification of some genes of T1, D1, and their parents.

(A) Transcriptome analysis results of D1, H3-2x and P1-2x materials; (B) In materials, the Transcriptome results of D1, H3-2x and P1-2x were verified by qPCR; (C) Transcriptome analysis results of T1, H3 and P1 materials; (D) qPCR validation of transcriptome results of T1, H3 and P1 materials. D1 is a diploid hybrid of P1-2x and H3-2x; T1 is a tetraploid hybrid of P1 and H3.


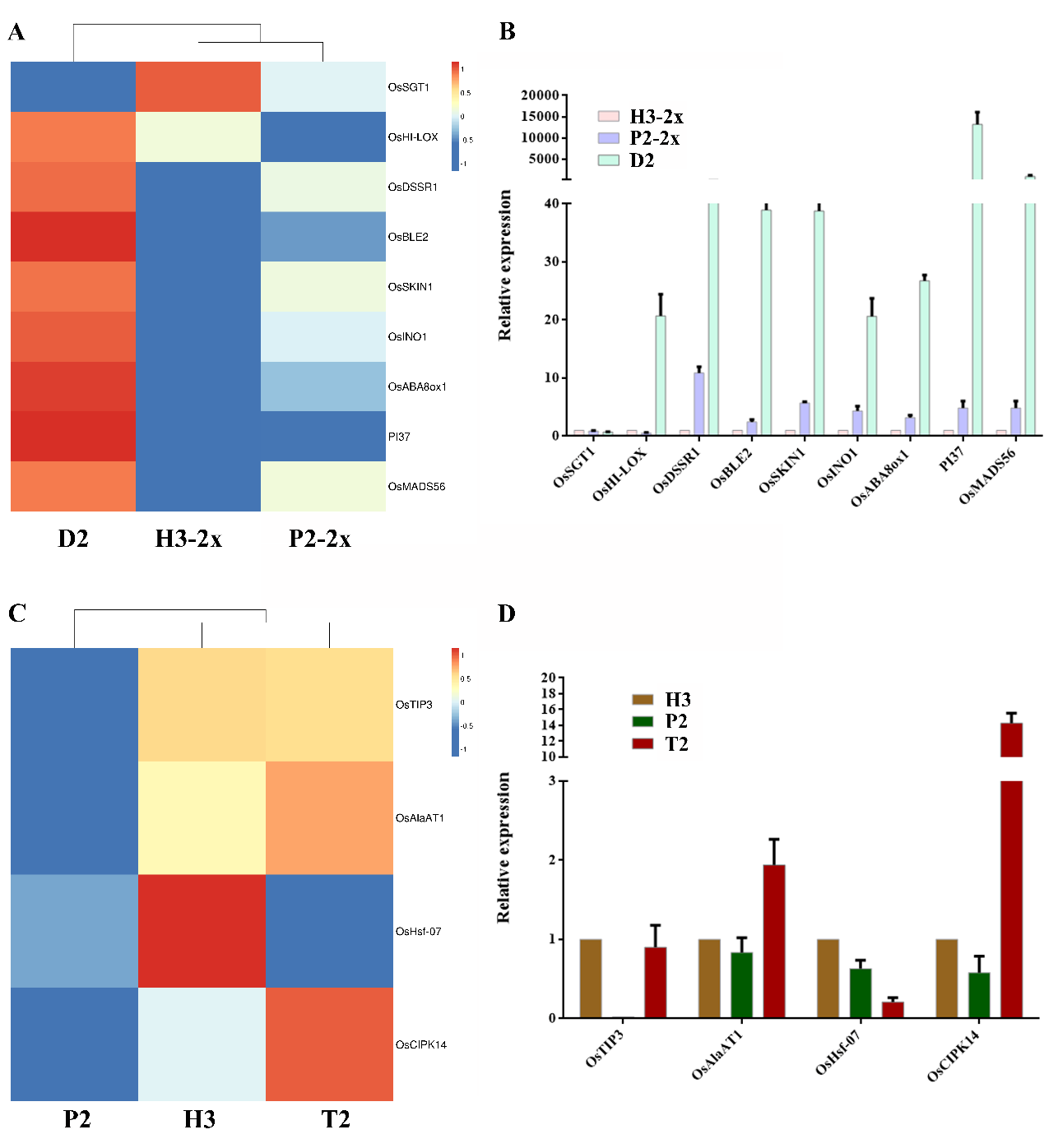


Figure S5 Transcriptome analysis and qPCR verification of some genes of T2, D2, and their parents.

(A) Transcriptome analysis results of D2, H3-2x and P2-2x materials; (B) In D2, H3-2x and P2-2x materials, the transcriptome results were verified by qPCR; (C) Transcriptome analysis results of T2, H3 and P2 materials; (D) In T2, H3 and P2 materials, the transcriptome results were verified by qPCR. D2 is a diploid hybrid of P2-2x and H3-2x; T2 is a tetraploid hybrid of P2 and H3.


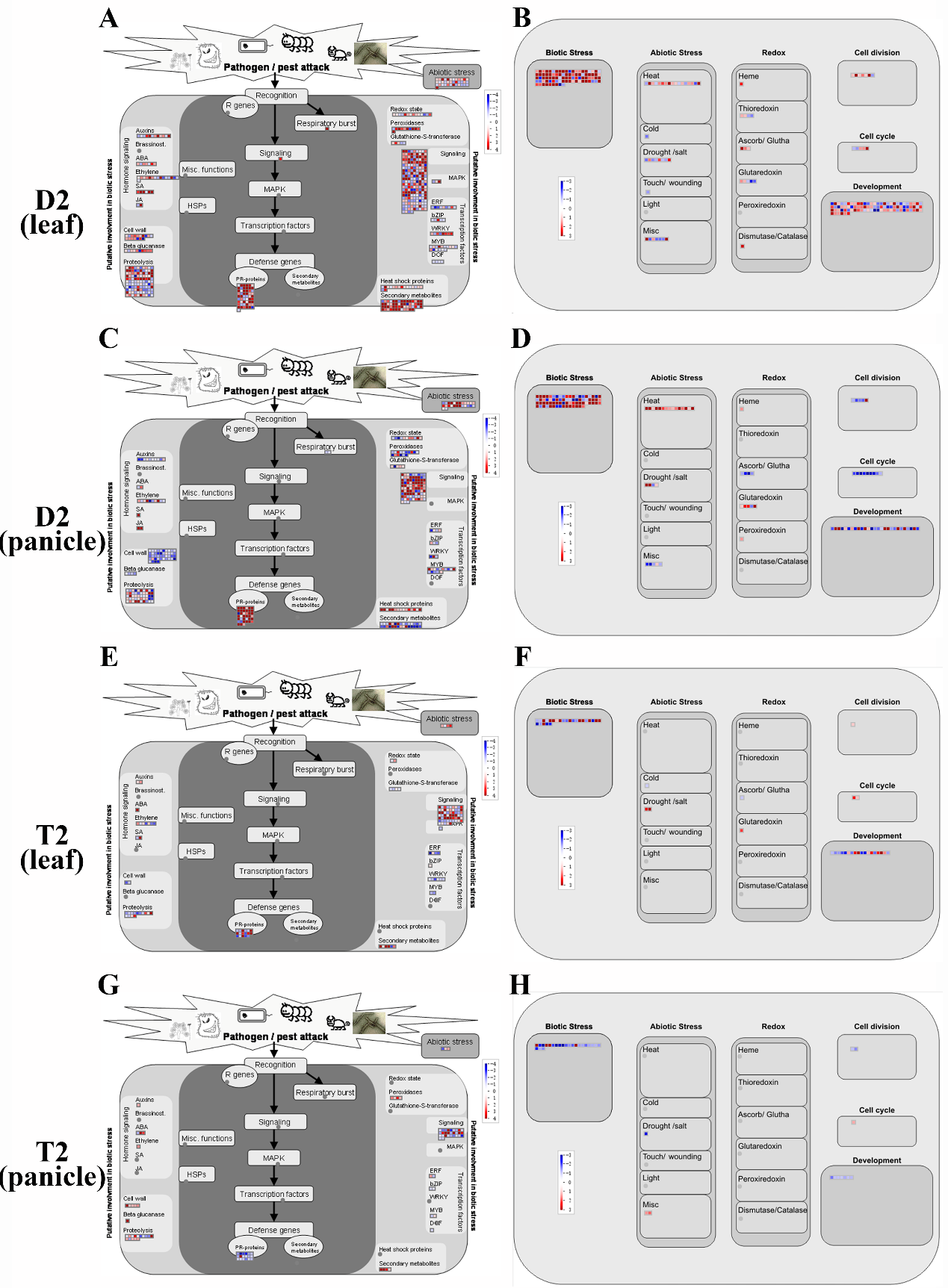


Figure S6 Path annotation of DEGFu-sp. 5 days after fertilization of D2 flag leaves; 5 days after fertilization of T2 flag leaves; 5 days after fertilization of D2 panicle; 5 days after fertilization of T2 panicle.

(A-B) representing differential gene set of DEGFu-sp-D2 about flag leaf; (C, D) indicating differential gene set of DEGFu-sp-D2 about panicle; (E-F) represent differential gene set of DEGFu-sp-T2 about flag leaf; (E-F) representing differential gene set of DEGFu-sp-T2 about flag leaf. The Mapping File is RAPDB-IRGSP1.0; D2: P2-2x × H3-2x; T2: P2 × H3.


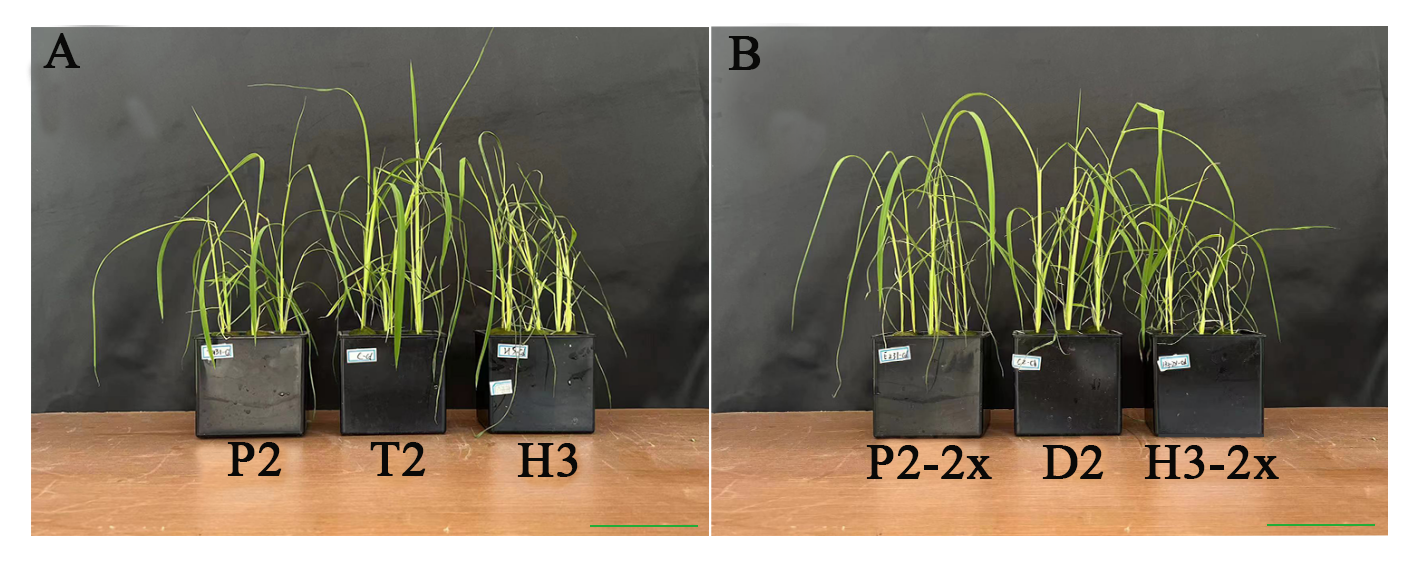


Figure S7 Phenotype of diploid (D2) and tetraploid (T2) rice hybrids and their parents under cadmium stress. The green bar is 11.5 cm. D2 is a diploid hybrid of P2-2x and H3-2x; T2 is a tetraploid hybrid of P2 and H3.


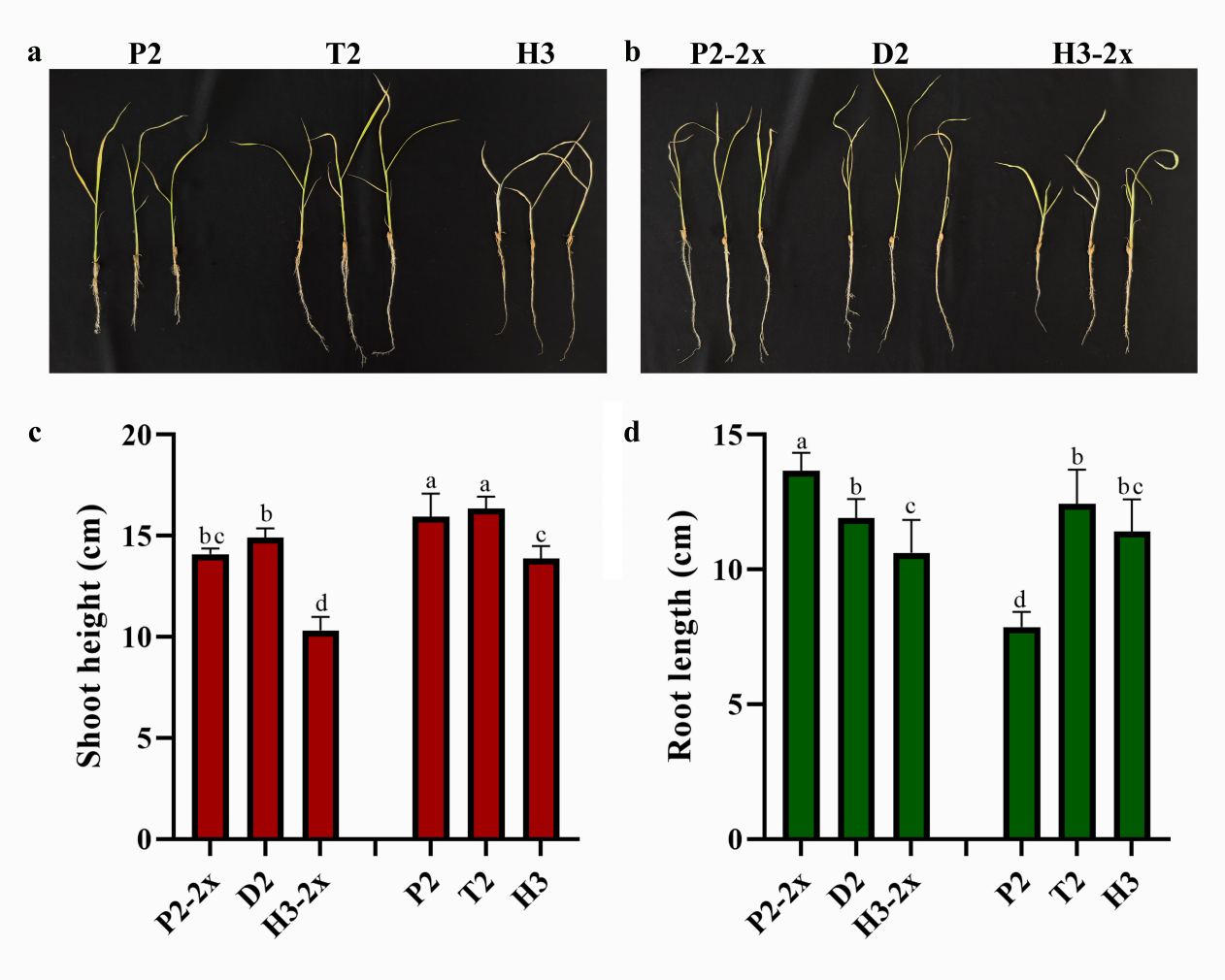


Figure S8 Shoot height and root length of diploid (D2) and tetraploid (T2) rice hybrids and their parents under salt stress. D2 is a diploid hybrid of P2-2x and H3-2x; T2 is a tetraploid hybrid of P2 and H3.





Figure S9 PCA, correlation, and Cohen’s d analysis for the phenotype data and different materials. CAR: carotenoid; Chl a: chlorophyll a; Chl b: chlorophyll b; H_2_O_2_: hydrogen peroxide; POD: peroxidase; CAT: catalase; Chl ab: chlorophyll ab; GSH-PX: glutathione; MDA: malondialdehyde; SOD: superoxide dismutase; D2 is a diploid hybrid of P2-2x and H3-2x; T2 is a tetraploid hybrid of P2 and H3.


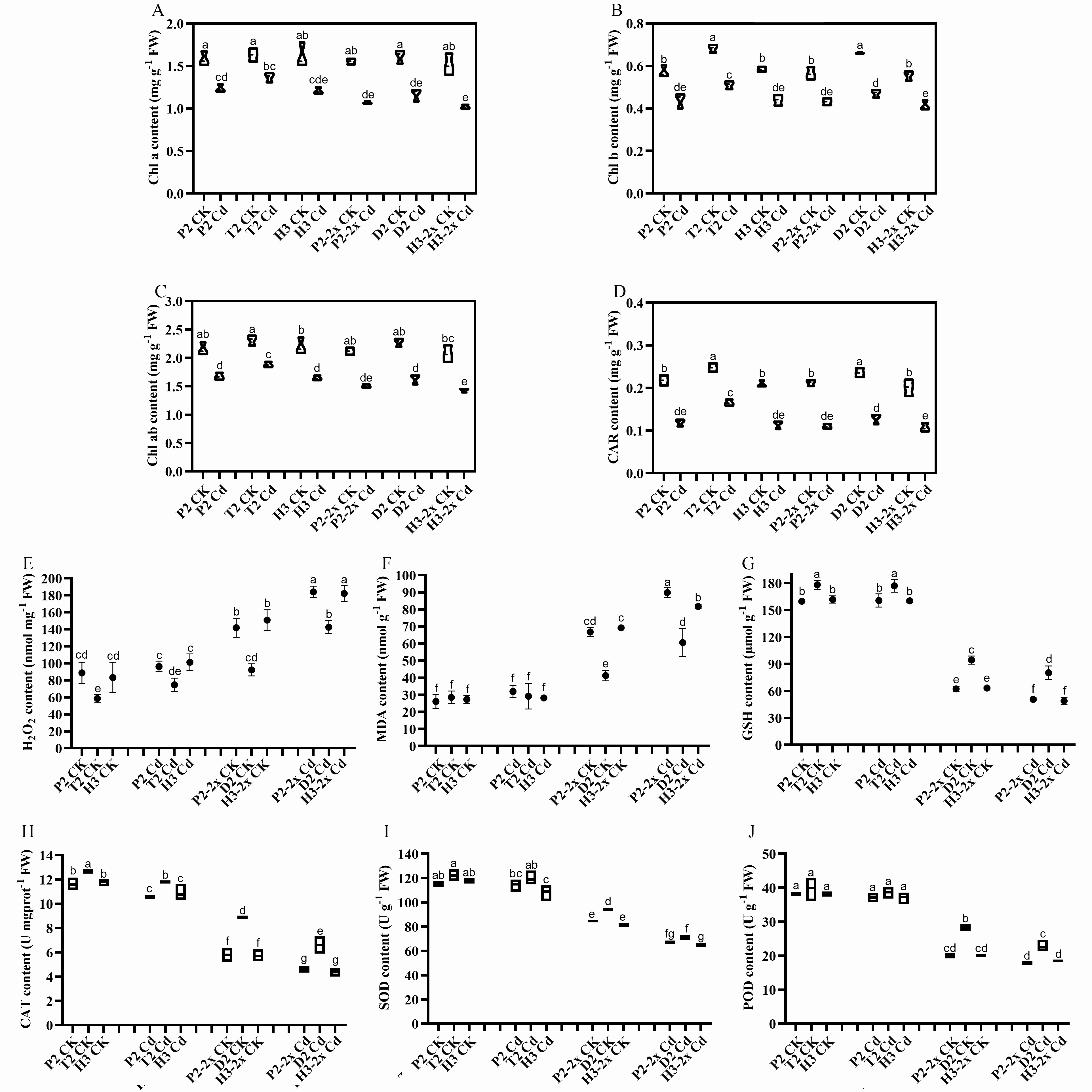


**Figure S10** Determination of physiological and biochemical indexes of diploid and tetraploid rice grown under cadmium-contaminated hydroponic conditions.

1. the content of Chl a in tetraploid and diploid hybrids and their parents; (B) the content of Chl b in tetraploid and diploid hybrids and their parents; (C) Chl ab content in tetraploid and diploid hybrids and their parents; (D) the content of CAR in tetraploid and diploid hybrids and their parents; (E) the content of H_2_O_2_ in tetraploid and diploid hybrids and their parents; (F) MDA content in tetraploid and diploid hybrids and their parents; (G) the contents of GSH in tetraploid and diploid hybrids and their parents; (H) the contents of CAT in tetraploid and diploid hybrids and their parents; (I) contents of total SOD in tetraploid and diploid hybrids and their parents; (J) contents of POD in tetraploid and diploid hybrids and their parents. D2 (P2-2x × H3-2x) and T2 (P2 × H3). Different small alphabetical letters above means revealed significant differences among treatments according to the LSD test (p < 0.05).


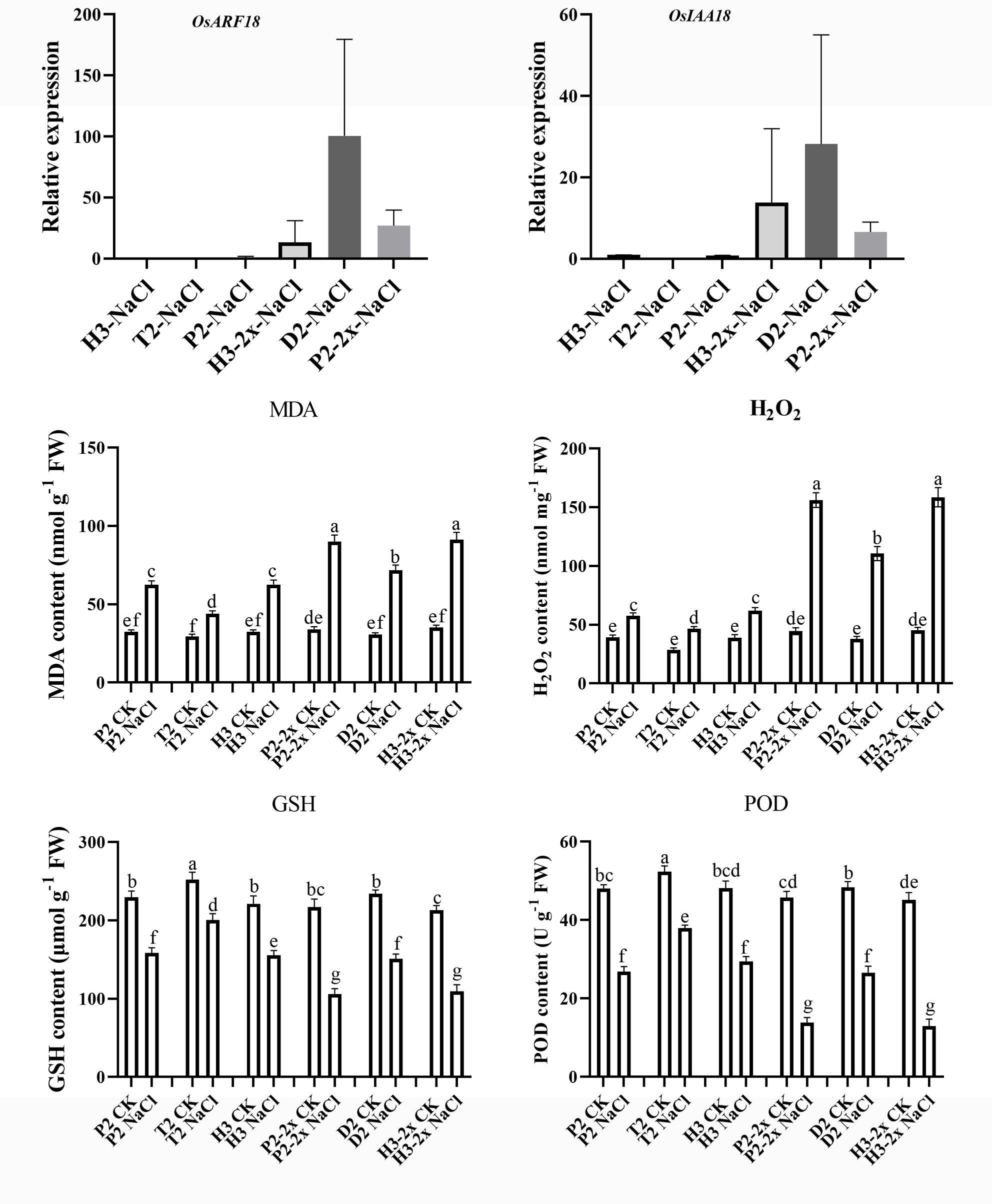


Figure S11 The expression patterns of *OsARF18* and *OsIAA18*, oxidative stress, enzymatic and non-enzymatic genes under salt stress; D2 is a diploid hybrid of P2-2x and H3-2x; T2 is a tetraploid hybrid of P2 and H3.


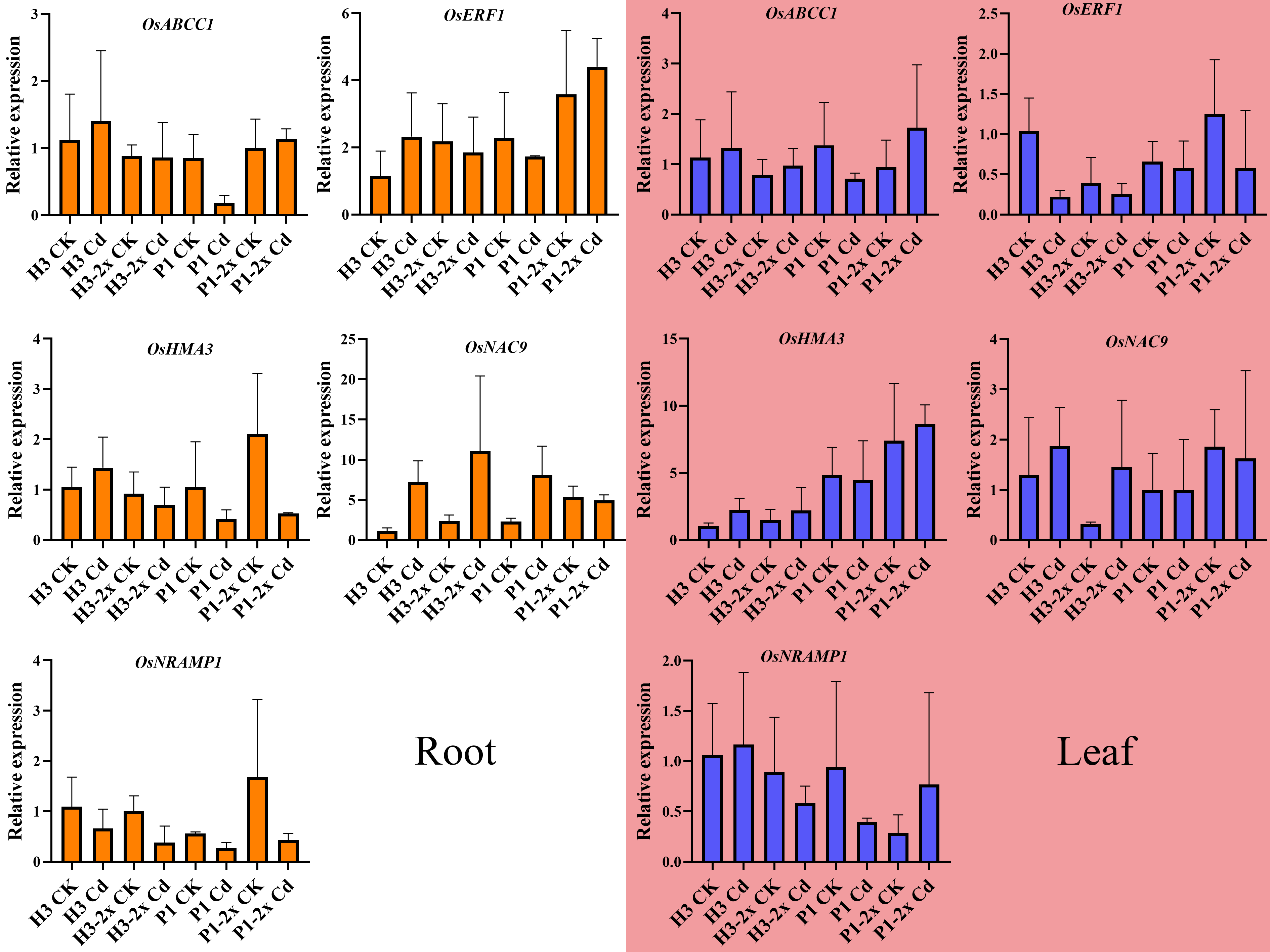


Figure S12 Expression of genes about Cd tolerance in root and shoot.

H3 is tetraploid rice; H3-2x is its diploid rice; P1 is autotetraploid rice; and P1-2x is its corresponding diploid rice. Ck and Cd indicate control and cadmium treatments.


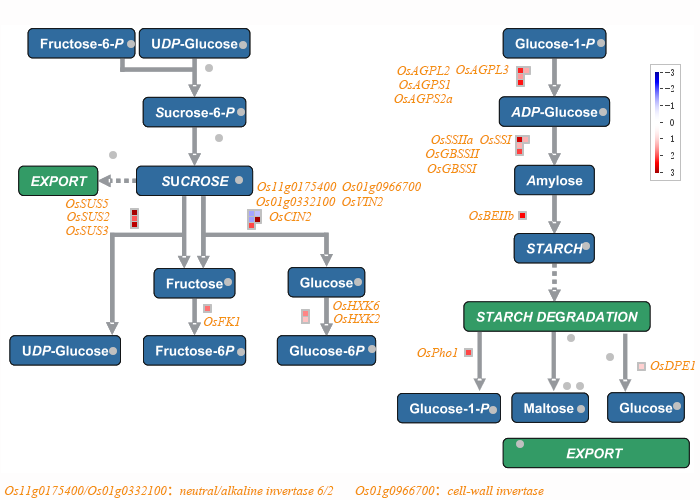


Figure S13 The process from sugar metabolism to starch synthesis.

*Os11g0175400/Os01g0332100:* neutral/alkaline invertase 6/2; *Os01g0966700*: cell-wall invertase


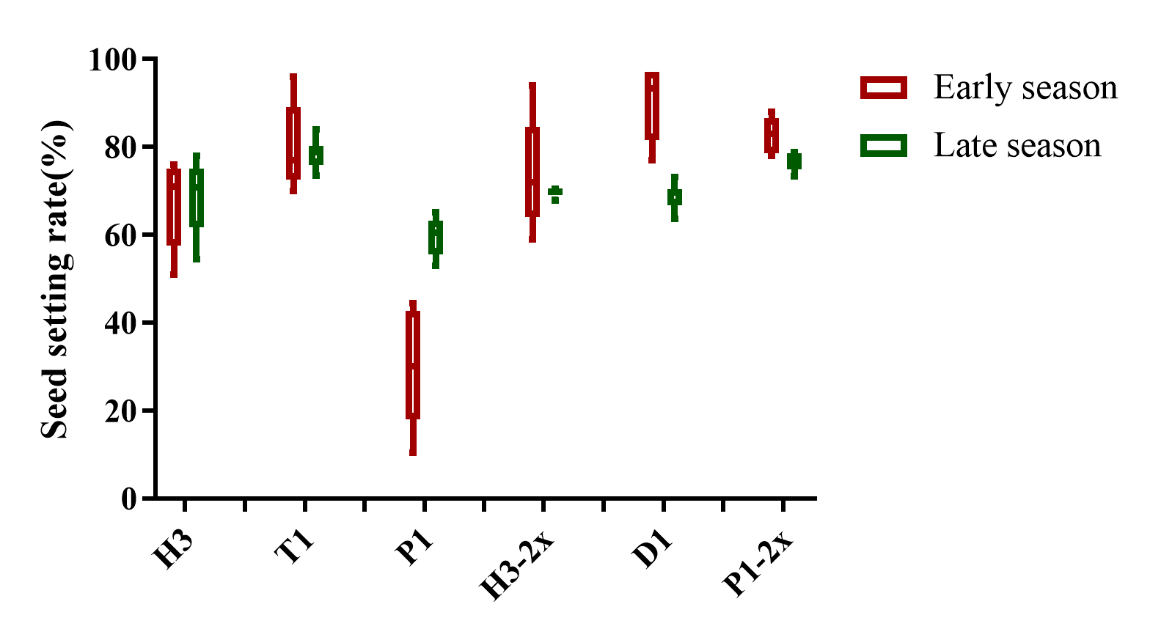


Figure S14 Seed setting of D1 and T1 hybrids, and their parental lines in early and late seasons.

D1 is a diploid hybrid of P1-2x and H3-2x; T1 is a tetraploid hybrid of P1 and H3.

**
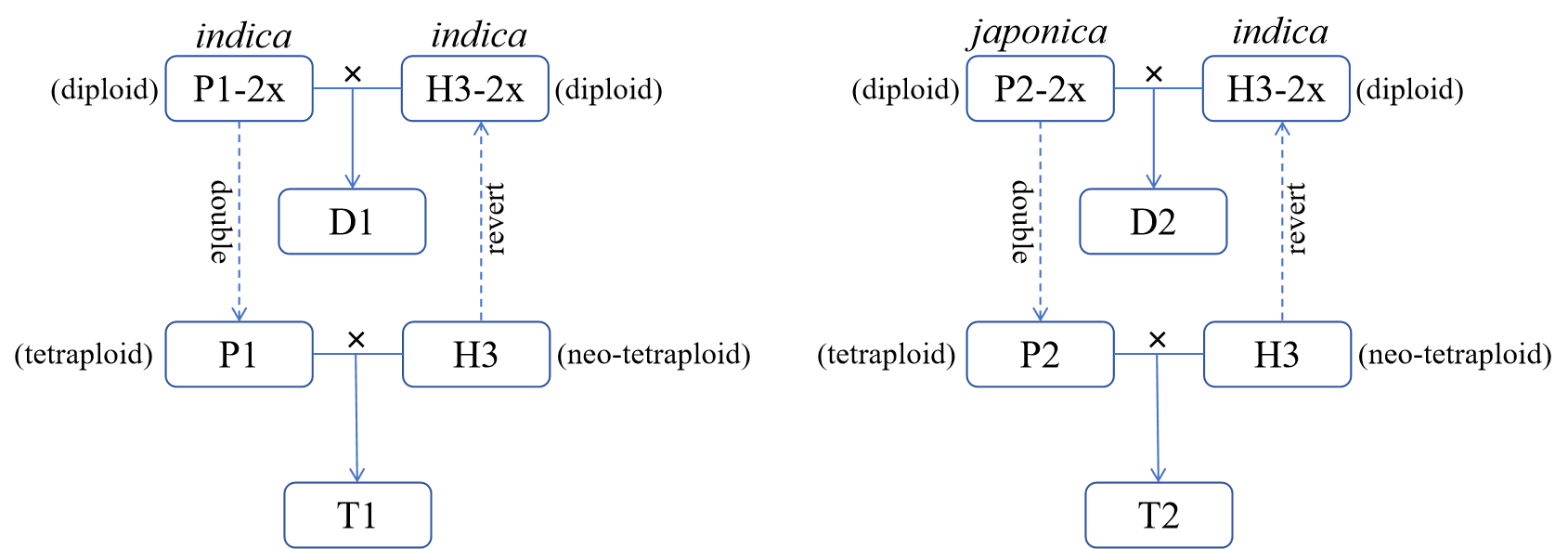
**

Figure S15 Diploid and tetraploid hybridization strategies.

D1 is a diploid hybrid of P1-2x and H3-2x; T1 is a tetraploid hybrid of P1 and H3; D2 is a diploid hybrid of P2-2x and H3-2x; T2 is a tetraploid hybrid of P2 and H3.
